# Interplay between transcriptional regulators and the SAGA chromatin modifying complex fine-tune iron homeostasis

**DOI:** 10.1016/j.jbc.2021.100727

**Published:** 2021-04-29

**Authors:** Manjit Kumar Srivastav, Neha Agarwal, Poonam Poonia, Krishnamurthy Natarajan

**Affiliations:** Laboratory of Eukaryotic Gene Regulation, School of Life Sciences, Jawaharlal Nehru University, New Delhi, India

**Keywords:** iron homeostasis, transcription factor, chromatin modification, gene regulation, *Candida albicans*, HAP complex, SEF1, CAP2, HAP43, SAGA complex, BPS, bathophenanthrolinedisulfonic acid, CoIP, coimmunoprecipitation, FAS, ferrous ammonium sulfate, +Fe, supplemented with FAS, −Fe, supplemented with BPS, SAGA, Spt-Ada-Gcn5-Acetyl transferase, SC-LIM, synthetic complete–low iron media

## Abstract

The human fungal pathogen *Candida albicans* responds to iron deprivation by a global transcriptome reconfiguration known to be controlled by the transcriptional regulators Hap43 (also known as Cap2), Sef1, and the trimeric Hap2-Hap3-Hap5 complex. However, the relative roles of these regulators are not known. To dissect this system, we focused on the *FRP1* and *ACO1* genes, which are induced and repressed, respectively, under iron deprivation conditions. Chromatin immunoprecipitation assays showed that the trimeric HAP complex and Sef1 are recruited to both *FRP1* and *ACO1* promoters. While the HAP complex occupancy at the *FRP1* promoter was Sef1-dependent, occupancy of Sef1 was not dependent on the HAP complex. Furthermore, iron deprivation elicited histone H3-Lys9 hyperacetylation and Pol II recruitment mediated by the trimeric HAP complex and Sef1 at the *FRP1* promoter. In contrast, at the *ACO1* promoter, the HAP trimeric complex and Hap43 promoted histone deacetylation and also limited Pol II recruitment under iron deprivation conditions. Mutational analysis showed that the SAGA subunits Gcn5, Spt7, and Spt20 are required for *C. albicans* growth in iron-deficient medium and for H3-K9 acetylation and transcription from the *FRP1* promoter. Thus, the trimeric HAP complex promotes *FRP1* transcription by stimulating H3K9Ac and Pol II recruitment and, along with Hap43, functions as a repressor of *ACO1* by maintaining a deacetylated promoter under iron-deficient conditions. Thus, a regulatory network involving iron-responsive transcriptional regulators and the SAGA histone modifying complex functions as a molecular switch to fine-tune tight control of iron homeostasis gene expression in *C. albicans*.

Living cells actively and efficiently maintain iron homeostasis, a process leading to the maintenance of optimal intracellular iron levels. The ability of pathogens to adapt to host environments is intimately linked to how gene expression is modulated by transcription factors and chromatin regulators. *Candida albicans*, the most prevalent human fungal pathogen, is an opportunistic pathogen and becomes virulent in immunocompromised host (1, 2). Iron is an essential micronutrient required for various metabolic steps, but has poor bioavailability in the mammalian host with vastly different concentrations in blood stream, gastrointestinal tract, and deep-seated tissues (3). Thus *C. albicans* has evolved multiple iron uptake strategies to mobilize iron from extracellular sources (reviewed in (4, 5, 6, 7, 8, 9)).

*C. albicans* cells respond to iron levels in the culture media by global transcriptome reconfiguration, involving upregulation of numerous genes including those in the multiple iron uptake pathways, and downregulation of genes encoding iron-requiring enzymes and proteins (10, 11, 12). This large transcriptional output is mediated by the transcriptional regulators Sef1 (11), HAP complex (13, 14, 15) and Cap2/HAP43 (14, 15), and the negative regulator Sfu1 (10, 11) and is summarized in Figure 1. Iron deprivation elicited the transcriptional induction of *SEF1*, leading to the induced expression of *HAP43*, *HAP2*, and *HAP3* genes (Fig. 1). The Hap2, Hap3, and Hap5 proteins form a trimeric HAP complex that binds to the CCAAT motif *in vitro* (16). The Hap43 transcriptional regulator bearing the amino terminal Hap4L-bZIP bipartite domain is associated with the trimeric HAP complex in cell extracts (14). Moreover, the Hap43 carboxyl terminal domain is essential for Hap43 transcriptional activity *in vivo* (17, 18). *C. albicans* mutants bearing deletions of *HAP2*, *HAP3*, *HAP5*, or *HAP43* genes led to misregulation of the expression of iron homeostasis genes and impaired cellular growth under iron deplete conditions (11, 13, 14, 15, 19). Sef1 is a Cys_6_Zn_2_ domain transcriptional regulator required for growth under Fe-limiting conditions (19). Upon iron deprivation, the *SEF1* mRNA expression is induced (11), and the Sef1 protein is localized to the nucleus (20) leading to the transcriptional induction of iron homeostasis genes (11, 14).

**Figure 1:**
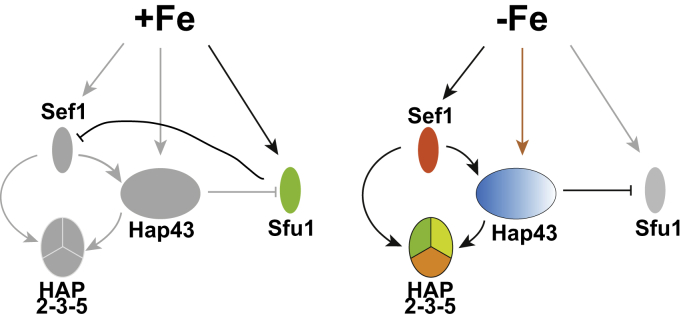
**Schematic representation of transcriptional control of iron-level responsive transcription factor genes in*****C. albicans*****.** The network of interdependent transcriptional control by Hap43, Hap2-Hap3-Hap5, Sef1, and Sfu1 under −Fe and +Fe is indicated. The symbols represent either active or induced (in *different colors*) or inactive or repressed (in *gray*) transcription factors. The *pointed arrows*, in *dark*, represent activation or interaction and *blunt arrows* depict inhibition or repression, while the *gray arrows* depict inactivity.

Genome-wide analyses showed that Hap43 and Sef1 have a widespread role in transcriptional control of iron homeostasis. Microarray analysis showed that of the Hap43-regulated genes, ∼56% were repressed and ∼44% induced were under Fe-deplete conditions (11, 14). Furthermore, among the genes that showed Sef1-dependent differential expression, ∼76% are induced and the expression of the rest are repressed under Fe-deplete conditions (11), suggesting that both Hap43 and Sef1 have dual regulatory functions. Interestingly, comparison of the Sef1 and Hap43 transcriptomes showed that ∼35% are induced and ∼14% repressed commonly in a Hap43-and Sef1-dependent manner (11, 14) indicating coregulation by these two TFs.

Genome-wide ChIP-Chip and microarray analyses showed that Sef1 is recruited to the promoters of ∼20% (∼60 genes) of low iron-induced genes including several iron uptake genes (for example, *FRP1* encoding ferric reductase) and to the promoters of *HAP2*, *HAP3*, and *HAP43* transcriptional regulator genes (11). The Hap2-Hap3-Hap5 trimeric complex, however, is recruited to both *FRP1* and *ACO1* gene promoters, respectively, to activate or repress their transcription under Fe-deprivation conditions (14). Although Hap43 controls the induction of ∼44% (224 genes) of the transcriptome, paradoxically, Hap43 is bound largely to low-iron repressed gene promoters (11). When iron is surfeit, Sfu1, a GATA-type transcriptional regulator binds to the promoters of several iron uptake genes including *FRP1* and that of the *SEF1* regulator to down regulate their expression (10).

Transcriptional control is intimately linked to the chromatin state (21). Acetylation of nucleosomal histone H3 at Lys9/14 is a transcriptionally active chromatin mark catalyzed by the SAGA histone acetyltransferase complex (22). SAGA is a large multifunctional protein complex that functions as a coactivator of transcription and is evolutionarily conserved from budding yeast to humans (23, 24), and in *C. albicans* (25). Several key aspects of the transcriptional regulation by the *HAP* genes and the *SEF1* gene are poorly understood. First, it is unclear how the *HAP* complex and the *SEF1* genes coordinate transcriptional activation of iron homeostasis gene expression. Second, the mechanism of how the HAP trimeric complex and the Hap43 mediate transcriptional repression of iron utilizing genes. In this study, we have identified the hierarchy of regulation of target genes by Sef1, HAP complex, and the Hap43 to orchestrate a combinatorial regulatory network resulting in modulation of SAGA-mediated histone acetylation and RNA polymerase (Pol) II recruitment, thereby providing tight transcriptional control of iron homeostasis.

## Results

### Interdependent expression of HAP complex subunits and Sef1

**Figure 2:**
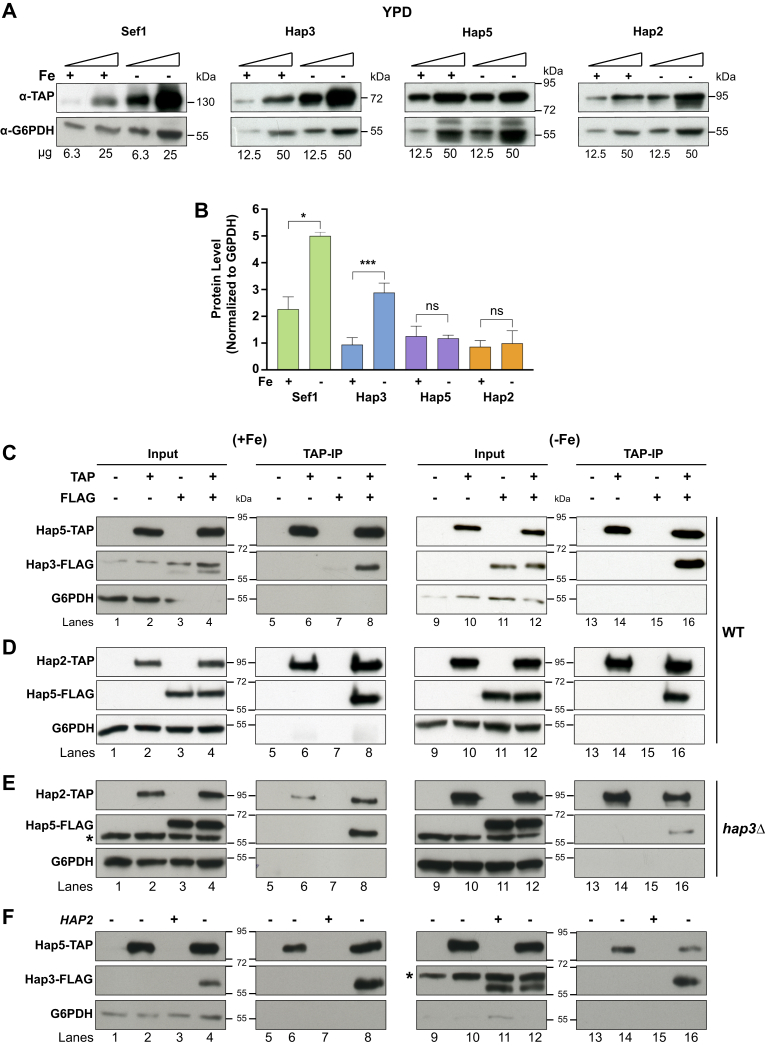
**Coimmunoprecipitation analysis revealed intersubunit interactions for HAP complex assembly.***A* and *B*, Quantitative western blot analysis of HAP complex subunits. Cell extracts from TAP-tagged strains RPY453 (Hap5), KNC398 (Hap2), KNC410 (Hap3), and KNC379 (Sef1) grown under iron replete (+Fe) and deplete (−Fe) conditions were analyzed by western blotting either with anti-TAP or with anti-G6PDH antibody as loading control, and images were quantified, normalized to G6PDH levels, and plotted as normalized protein expression. Note that the images of Sef1 and G6PDH panels in (*A*) are from the same experiment but of different exposure times. The error bars represent SEM (n = 4). *C*–*E*, Hap3 is critical for HAP trimeric complex formation under −Fe conditions. *C. albicans* whole-cell extracts prepared from untagged (SN152), TAP-tagged strains, or FLAG-tagged strains grown under iron-replete or -deplete conditions were used. *C*, coimmunoprecipitation of Hap5-TAP and Hap3-FLAG in WT background. Western blot analysis of input (lanes 1–4, 9–12) and IP lanes (lanes 5–8, 13–16) for untagged strain SN152 (lanes 1, 5, 9, 13), strain RPY453 (Hap5-TAP; lanes 2, 6, 10, 14), KNC691 (Hap3-FLAG; lanes 3, 7, 11, 15) and KNC692 (Hap5-TAP and Hap3-FLAG; lanes 4, 8, 12, 16). *D*, coimmunoprecipitation of Hap2-TAP and Hap5-FLAG in WT background. Western blot analysis of input (lanes 1–4, 9–12) and IP lanes (lanes 5–8, 13–16) for strain SN152 (lanes 1, 5, 9, 13), strain KNC398 (Hap2-TAP; lanes 2, 6, 10, 14), RPY552 (Hap5-FLAG; lanes 3, 7, 11, 15), and KNC553 (Hap2-TAP and Hap5-FLAG; lanes 4, 8, 12, 16). *E*, coimmunoprecipitation of Hap2-TAP and Hap5-FLAG in *hap3Δ/Δ* mutant background. Western blot analysis of input (lanes 1–4, 9–12) and IP lanes (lanes 5–8, 13–16) for strain RPY431 (lanes 1, 5, 9, 13), strain KNC560 (Hap2-TAP; lanes 2, 6, 10, 14), KNC555 (Hap5-FLAG; lanes 3, 7, 11, 15), and KNC558 (Hap2-TAP and Hap5-FLAG; lanes 4, 8, 12, 16). *F*, coimmunoprecipitation of Hap5-TAP and Hap3-FLAG in *hap2Δ/Δ* mutant background. Western blot analysis of input (lanes 1–4, 9–12) and IP lanes (lanes 5–8, 13–16) for untagged strain TF087 (lanes 1, 5, 9, 13), strain KNC454 (Hap5-TAP; lanes 2, 6, 10, 14), KNC691 (Hap3-FLAG; lanes 3, 7, 11, 15), and KNC693 (Hap5-TAP and Hap3-FLAG; lanes 4, 8, 12, 16). A nonspecific band obtained in all input samples with anti-FLAG antibody is indicated with an *asterisk* (∗).

To investigate the steady-state protein levels of each of the HAP complex and Sef1 transcriptional regulators, we TAP-tagged the respective genes at their endogenous loci and carried out quantitative western blot analysis of cell extracts prepared from cells grown in two different media—either SC-LIM or the YPD media containing BPS (−Fe) or BPS plus FAS (+Fe). Quantification of the western blot data showed that although Hap2, Hap3, Hap5, and Sef1 are all expressed under both −Fe and +Fe conditions, only Hap3 and Sef1 proteins were upregulated (∼3.5-fold) in the BPS-containing YPD (Fig. 2, *A* and *B*) and SC-LIM media (Fig. S1, *A* and *B*). The elevated Sef1 and Hap3 protein levels in −Fe medium (Fig. 2, *A* and *B*) correlated well with the upregulation of the cognate mRNAs (11, 14). Although *HAP2* mRNA was slightly induced (11, 14), our western blot data did not reveal an increase in steady-state level of the Hap2 protein in −Fe compared with +Fe conditions.

To determine if *HAP43* expression is controlled at the level of transcription, we constructed a *HAP43-lacZ* promoter fusion and analyzed expression in WT, *hap43*Δ, and *sef1*Δ mutants. The reporter fusion was activated by approximately 6-fold in WT strain in −Fe medium (Fig. S2*A*), and this activation was not impaired in the *hap43*Δ mutant indicating that *HAP43* expression is not controlled by Hap43. In the *sef1Δ* mutant, however, the activation of *HAP43-lacZ* reporter was only partially reduced (approximately 2-fold) compared with the WT level (Fig. S2*A*). Therefore, we quantified *HAP43* mRNA level in −Fe *versus* +Fe condition and found that the mRNA is induced ∼6-fold in WT, but the induction was lost in the *sef1*Δ mutant cells (Fig. S2*B*). Western blot analysis showed that although Hap43 protein level was also substantially reduced (Fig. S2, *C* and *D*), the Hap43 protein was still detectable in *sef1*Δ/Δ mutant cell extracts, indicating Sef1-independent partial expression of *HAP43* in iron-starved cells.

To examine the requirement of the HAP subunits for HAP complex formation, we conducted CoIP assays using epitope-tagged Hap5, Hap2, and Hap3 in WT, *hap3Δ/Δ*, or *hap2Δ/Δ* strain backgrounds. In each case, we used TAP-tagged subunits to CoIP and probe the western blots with anti-FLAG antibody to identify protein–protein interactions. The data showed that Hap5 strongly pulled down Hap3 from WT cell extracts grown in −Fe medium, but not from +Fe cell extracts (lanes 8 and 16; Fig. 2*C*), likely due to the diminished expression of the Hap3 protein in +Fe compared with −Fe (Fig. 2*B*). The Hap2 protein was efficiently coimmunoprecipitated by Hap5 from cell extracts grown in +Fe or −Fe media (lanes 8 and 16; Fig. 2*D*). Moreover, the Hap2-Hap5 interaction was greatly impaired by the *hap3Δ* mutation in −Fe but not in +Fe condition (lanes 8 and 16; Fig. 2*E*). The CoIP data also showed that Hap5 interacts with Hap3 in both −Fe and +Fe cell extracts that is largely unaffected by *hap2*Δ/Δ mutation (Fig. 2*F*). Together these CoIP data indicated that the induced expression of Hap3 under −Fe condition is essential for Hap5-Hap2 interaction and assembly of Hap3 to form the HAP trimeric complex under iron starved condition. In +Fe condition, however, Hap5 interacted with Hap2 despite the diminished Hap3 protein level, thus indicating Hap3-independent interaction of Hap5 and Hap2.

### Selectivity of promoter occupancy by the HAP trimeric complex subunits and Sef1

To address how the functions of these transcriptional regulators impinge on iron-responsive promoters, we carried out ChIP assays to confirm the *in vivo* occupancy of each subunit of the HAP complex and the Sef1 regulators. Our previous studies showed that under iron deprivation conditions Hap5 was recruited to *FRP1* promoter (−84 to −281 with respect to ATG) and *ACO1* promoter (−201 to −340) bearing the CCAAT motif (14). Therefore, we chose these promoters to analyze the occupancy of Hap5, Hap2, Hap3, Hap43, and Sef1 under iron deplete and iron-replete conditions using TAP-tagged *C. albicans* strains. Because *HAP43* lost its activity upon epitope tagging (14), we used affinity-purified polyclonal anti-Hap43 antibody described previously (14) for the ChIP assay.

**Figure 3:**
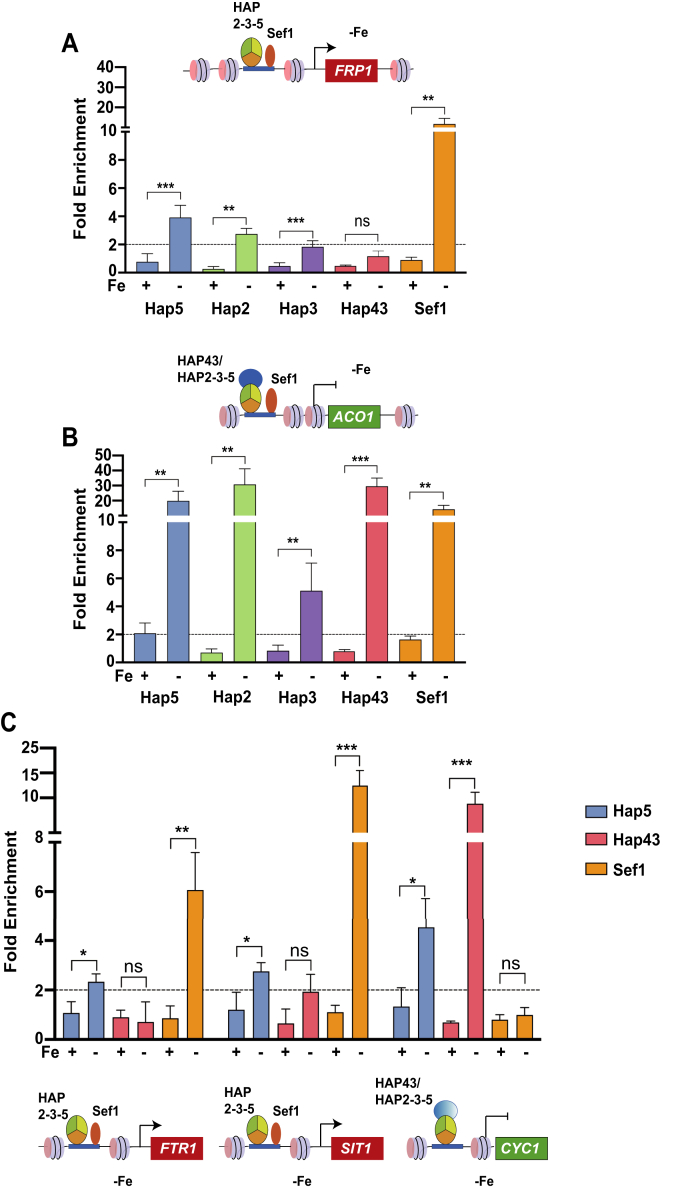
**Differential occupancy of trimeric HAP complex, Hap43, and Sef1 at iron homeostasis gene promoters.***A* and *B*, ChIP analysis of the occupancy of Hap2, Hap3, Hap5, Hap43, and Sef1 TFs at (*A*) *FRP1* and (*B*) *ACO1* gene promoters. Cells were cultured in SC- LIM medium with BPS plus FAS (+Fe) or with BPS alone (−Fe), cross-linked with formaldehyde, cells were lysed and sonicated to prepare chromatin extracts. The TAP-tagged proteins in chromatin extracts from strains Hap5-TAP (RPY453), Hap2-TAP (KNC398), Hap3-TAP (KNC410). and Sef1-TAP (KNC379) and the untagged strain as control were purified by IgG-Sepharose, and the *FRP1* and *ACO1* promoter regions and the control Chr I region were analyzed by real-time qPCR. The specific enrichment of the tagged proteins was calculated with reference to the background enrichment in untagged extract, and the relative fold enrichment of the specific promoter region normalized to the nonspecific Chr I region was plotted. Hap43 ChIP analysis was conducted in WT strain or from control isogenic *hap43Δ/Δ* mutant strain using affinity purified anti-Hap43 antibody and *FRP1* and *ACO1* promoters were analyzed by qPCR. The specific enrichment of Hap43-bound promoters with reference to the background enrichment in the *hap43Δ* mutant and the relative fold enrichment were calculated by normalization to the nonspecific Chr I region. *C*, ChIP analysis to assess recruitment of the Hap5, Hap43, and Sef1 at the iron homeostasis gene promoters *FTR1*, *SIT1*, and *CYC1* under +Fe and −Fe conditions as described above. Statistical analysis for specific enrichment in +Fe compared with −Fe was calculated using Student's *t*-test in GraphPad Prism 6, and the error bars represent SEM (n = 3). *p*-value ≤ 0.05 (∗), ≤0.01 (∗∗), ≤0.001 (∗∗∗), and >0.05 (ns).

Our ChIP data showed that the occupancy of each of the three components of the HAP trimeric complex, Hap5, Hap2, and Hap3 is enhanced at the *FRP1* promoter (Fig. 3*A*) and at the *ACO1* promoter (Fig. 3*B*) in −Fe condition compared with +Fe condition. We also extended the analyses to other low-iron-induced promoters (*FTR1*, *SIT1*) as well as to another low-iron-repressed promoter (*CYC1*) using the same ChIP samples. As expected, occupancy of Hap5 was elevated at all three promoters under −Fe compared with +Fe condition (Fig. 3*C*). While Hap43 occupancy was undetectable at the *FRP1* promoter (Fig. 3*A*), its occupancy was substantially high at the *ACO1* promoter (Fig. 3*B*) and the *CYC1* promoter under Fe-deplete condition (Fig. 3*C*). The ChIP analysis also showed Sef1 occupancy at *FRP1*, *FTR1*, *SIT1*, and *ACO1* promoters but not to the *CYC1* promoter (Fig. 3*C*). Given that Sef1 is a transcriptional activator, it was surprising to detect Sef1 occupancy at the *ACO1* promoter under −Fe condition. Indeed, previous ChIP-chip data also seemed to identify Sef1 binding to the promoters of *ACO1*, *ISA1*, *HEM3*, and *SEF2* genes whose expression is repressed under iron-deficient condition (11), although the significance of this data was not clear.

Together these ChIP data confirmed that the HAP trimeric complex is recruited to low-iron-induced iron uptake gene promoters as well as to the low-iron-repressed iron utilizing gene promoter under iron deprivation conditions. Hap43, however, showed a robust recruitment only to the low-iron-repressed promoters. Furthermore, Sef1 occupancy to low-iron-induced promoters (*FRP1, FTR1*, and *SIT1*) was elevated and, surprisingly, to the low-iron-repressed *ACO1* promoter but not to the *CYC1* promoter. Thus, iron deprivation elicited the induction of HAP trimeric complex occupancy at both iron uptake and iron utilizing gene promoters. Furthermore, iron deprivation also elicited the induction of Sef1 and Hap43 occupancy at the iron uptake and the iron utilizing gene promoters, respectively.

### Interdependency of the HAP complex and Sef1 regulators for promoter occupancy

**Figure 4:**
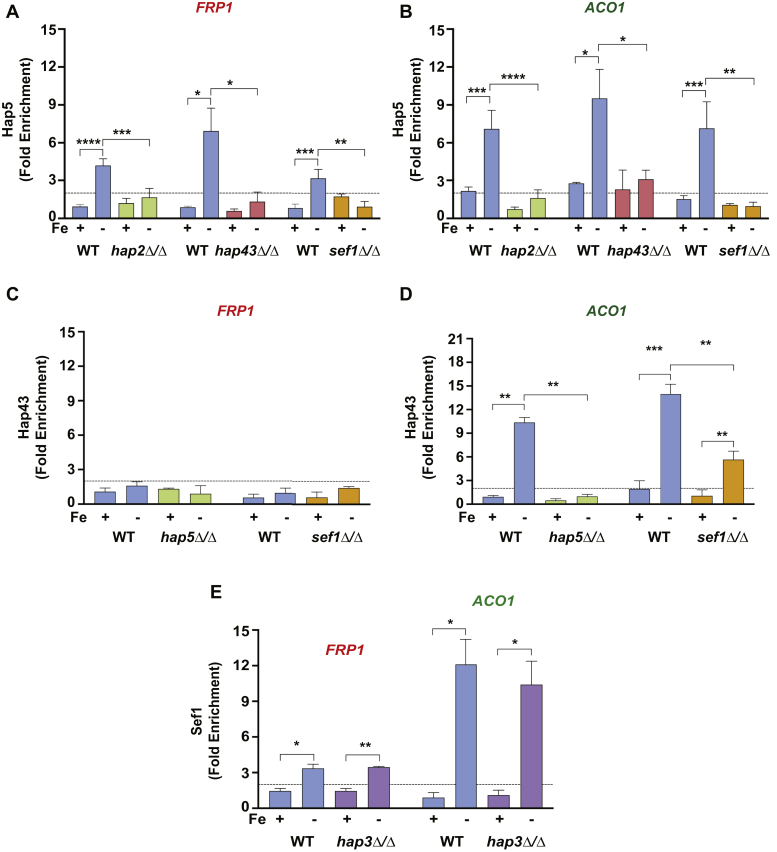
**Recruitment of Hap5 and Hap43 to iron homeostasis gene promoters is interdependent on each other and on Sef1.***A* and *B*, ChIP analysis of Hap5-TAP occupancy at the *FRP1* or *ACO1* promoters in WT (RPY453), *hap2Δ/Δ*(KNC454), *hap43Δ/Δ* (RPY471), or *sef1Δ/Δ* (KNC430) strains grown in +Fe and −Fe containing media. *C* and *D*, ChIP analysis of Hap43 occupancy at *FRP1* and *ACO1* promoters in WT (SN152), *hap5Δ/Δ*(TF093), or *sef1Δ/Δ* (TF015) strains under +Fe and −Fe containing media. *E*, ChIP analysis of Sef1-TAP in wild-type (WT; KNC369) and *hap3Δ/Δ*(KNC402) strains. Statistical analysis for specific enrichment in +Fe compared with −Fe was calculated using Student's *t*-test in GraphPad Prism 6, and the error bars represent SEM (n = 3). *p*-value ≤ 0.05 (∗), ≤0.01 (∗∗), ≤0.001 (∗∗∗), ≤0.0001 (∗∗∗∗), and >0.05 (ns).

Next, we asked if the HAP trimeric complex, Sef1, and Hap43 transcriptional regulators are mutually dependent for the recruitment of each regulator to promoters *in vivo*. We constructed Hap5-TAP expressing strains in WT or in *hap2*Δ/Δ, *hap43Δ/Δ*, or *sef1Δ/Δ* mutant backgrounds, and Sef1-TAP strain in the background of *hap3Δ/Δ* mutation, and carried out ChIP assays with either IgG-Sepharose beads or affinity purified anti-Hap43 antibody. The ChIP data revealed that the recruitment of Hap5 to both *FRP1* (Fig. 4*A*) and *ACO1* promoters (Fig. 4*B*) in −Fe medium was abolished in the *hap2Δ/Δ* mutant indicating that Hap2 is required for Hap5 promoter occupancy. Our previous study showed that Hap5 recruitment is also dependent on Hap3 (14), thereby showing that Hap5, Hap2, and Hap3 function together in promoter binding of the HAP trimeric complex.

Next, to address the requirement of Sef1 for recruitment of the HAP complex, we conducted ChIP assays in *sef1Δ/Δ* strain expressing *HAP5-TAP*. The ChIP-qPCR results demonstrated that the Hap5 recruitment to both *FRP1* and *ACO1* promoters was lost in the *sef1*Δ/Δ mutant (Fig. 4, *A* and *B*). Furthermore, deletion of *hap43*Δ/Δ also abolished Hap5 recruitment at *ACO1* and *FRP1* promoters in Fe-deplete condition (Fig. 4, *A* and *B*). Together these data demonstrated that the HAP complex recruitment is highly dependent on both Hap43 and Sef1.

To address the requirement of the HAP trimeric complex and Sef1 for Hap43 recruitment, we conducted Hap43 ChIP analysis in *hap5*Δ/Δ and *sef1*Δ/Δ mutant strain backgrounds. The ChIP data showed that whereas Hap43, as expected, is not recruited to *FRP1* (Fig. 4*C*), its recruitment to *ACO1* promoter is completely dependent on the Hap5 subunit of the HAP trimeric complex (Fig. 4*D*). Next, we investigated the requirement of Sef1 for Hap43 recruitment to the *ACO1* promoter. The *sef1Δ/Δ* mutation did not abolish Hap43 recruitment to the *ACO1* promoter but was only reduced by ∼2.5-fold (Fig. 4*D*) indicating that Sef1 is not essential but required for full potential of Hap43 recruitment to promoter *in vivo*, consistent with partial dependence of *HAP43* mRNA expression on Sef1 (Fig. S2*D*).

To assess if the HAP complex is required for Sef1 recruitment, we carried out ChIP assays using TAP-tagged *SEF1* in WT and *hap3*Δ/Δ strain backgrounds. This experiment showed that Sef1 occupancy at the *FRP1* promoter, as shown above, was stimulated upon iron deprivation, but interestingly the *hap3*Δ/Δ mutation did not impair the Sef1 occupancy in Fe-depleted medium (Fig. 4*E*). Furthermore, the Sef1 occupancy at the *ACO1* promoter was also independent of Hap3 (Fig. 4*E*), indicating that Sef1 is recruited independent of the HAP complex at both *FRP1* and *ACO1* promoters.

### HAP complex occupancy is dependent on both HAP- and Sef1-binding sites

**Figure 5:**
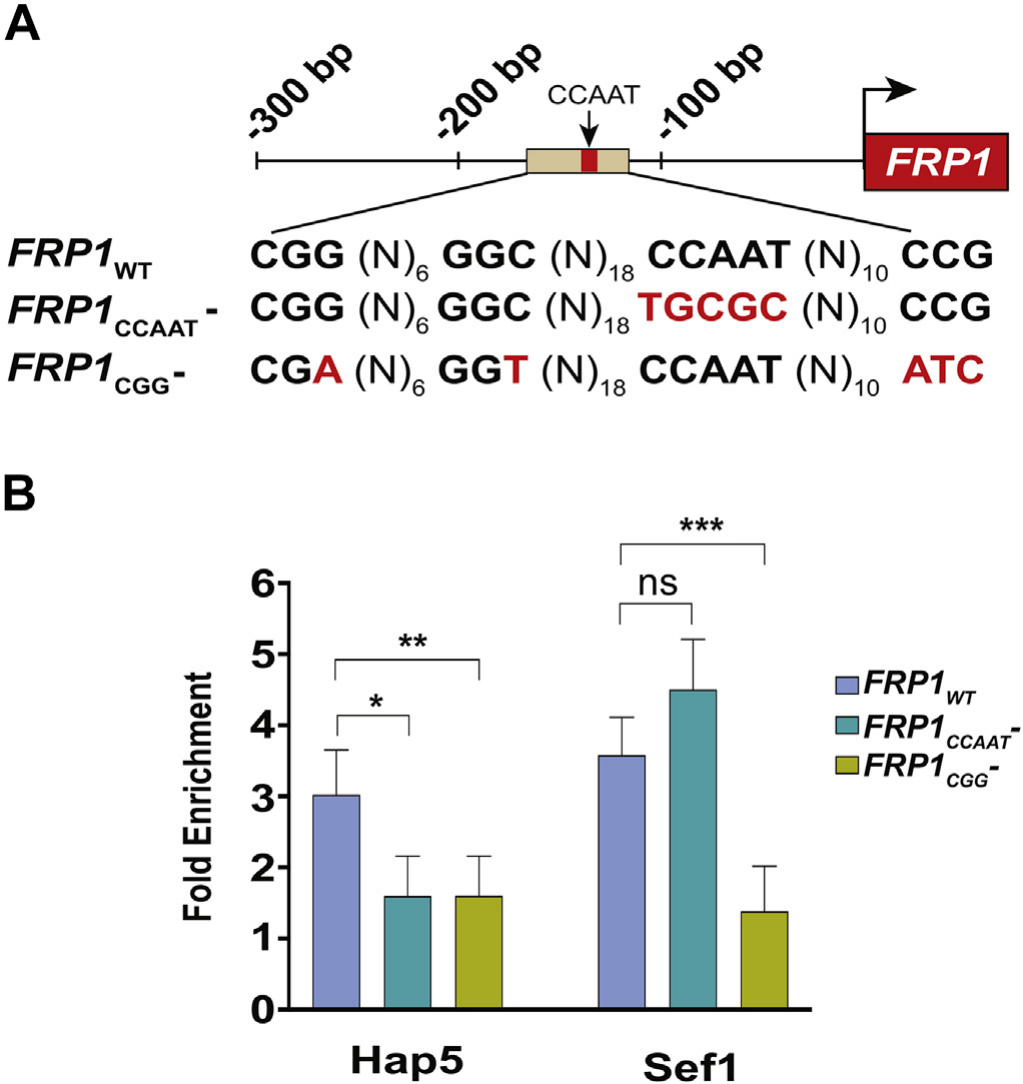
**HAP complex occupancy at the *FRP1* promoter is strongly dependent on Sef1 binding, but Sef1 occupancy is not dependent on HAP complex binding.***A*, schematic representation of the *FRP1* WT promoter showing the HAP complex binding site (CCAAT box) and 5′CGG, the putative Sef1-binding site. Mutations were introduced into the CCAAT box (*FRP1* CCAAT¯) and the CGG motif (*FRP1* CGG¯) in the *FRP1* promoter by site-directed mutagenesis and the nucleotide substitutions are indicated in *red*. *B*, Hap5 occupancy is strongly dependent on Sef1, but Sef1 occupancy is not Hap5-dependent. ChIP assays were conducted to analyze Hap5 occupancy at the WT *FRP1* promoter (in strain KNC479), the *FRP1* CCAAT¯ (in strain KNC494), or the *FRP1* CGG¯ (in strain KNC505) mutant promoters. Similarly, Sef1 occupancy was analyzed at the WT *FRP1* promoter (KNC486), the *FRP1* CCAAT¯(KNC498), or the *FRP1* CGG¯ (KNC509) mutant promoters in strains bearing TAP-tagged Sef1. The error bars represent SEM (n = 4). *p*-value ≤ 0.05 (∗), ≤0.01 (∗∗), ≤0.001 (∗∗∗), and >0.05 (ns).

To determine how Sef1 and the HAP complex mediate transcriptional activation, we chose the *FRP1* promoter since both Sef1 and the HAP complex are recruited to the same location in the *FRP1* promoter (Fig. 3*A*). The *FRP1* promoter contains the CCAAT motif at −134 with reference to the *FRP1* start codon, and this motif was shown to be critical for Hap5 binding and regulation of *FRP1* expression in response to iron deprivation (9). Although the putative binding site for Sef1 was proposed based on ChIP-Chip analysis (7), the exact sequence/s has not been experimentally demonstrated. Therefore, we looked for a putative Sef1 site, bearing a 5′CGG motif at the *FRP1* promoter and identified an elaborate sequence 5′CGG(N_6_)GGC(N_18_)CCAAT(N_10_)CCG with two apparent 5′CGG motifs in opposite orientations beginning at position −164 that nested the CCAAT motif (Fig. 5*A*).

To test the functional significance of the 5’CCAAT motif *in vivo*, we cloned the *FRP1* promoter and mutated the 5’CCAAT sequence to 5’TGCGC by site-directed mutagenesis. As the *in vivo* requirement of the 5’CGG motif was not known, we introduced mutations in the two 5’CGG motifs and constructed *FRP1* knock-in strains bearing either WT as control or those bearing point mutations within the CCAAT and CGG motifs (Fig. 5*A*) and examined the occupancy of Sef1 and HAP complex. The ChIP analysis showed Hap5 occupancy at the WT *FRP1* promoter in the knock-in cells in −Fe medium (Fig. 5*B*). However, the ChIP data also showe′d that the 5′CCAAT^–^ mutation impaired Hap5 occupancy to *FRP1* promoter *in vivo* (Fig. 5*B*), consistent with the requirement of the 5′CAAT motif for Hap5 binding to *FRP1* promoter *in vitro* reported previously (13).

Next, we conducted ChIP assays to examine Sef1 recruitment and found robust Sef1 recruitment to the WT *FRP1* sequence under iron-deplete condition (Fig. 5*B*). But the CGG^–^ mutation abrogated Sef1 occupancy (Fig. 5*B*), indicating the requirement of this promoter sequence for Sef1 binding to chromatin *in vivo*. Surprisingly, Hap5 occupancy was also impaired by the 5′CGG^–^ mutation (Fig. 5*B*) suggesting that a WT 5′CGG motif also promotes HAP complex binding. Interestingly, Sef1 occupancy was not impaired by the 5′CCAAT^–^ mutation (Fig. 5*B*), suggesting that Sef1 occupancy at *FRP1* promoter *in vivo* occurs independent of the 5′CCAAT motif. Together these data suggest that the HAP complex recruitment is dependent on Sef1 occupancy but recruitment of Sef1 seemed to be independent of the HAP complex occupancy at the *FRP1* promoter *in vivo*.

### Modulation of H3K9 acetylation by HAP complex and Sef1 at promoters

**Figure 6:**
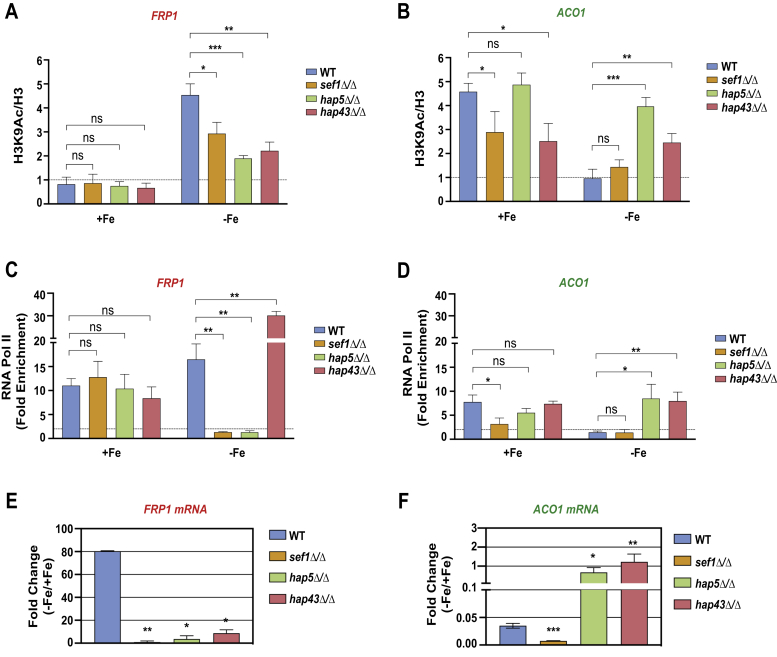
**Promoter-level histone H3-K9 acetylation is stimulated by Sef1 and the HAP complex.** ChIP assay was used to measure the level of histone H3-K9 acetylation at (*A*) *FRP1* and (*B*) *ACO1* promoters in the wild type (WT; SN152), or the *sef1Δ/Δ*(TF015), *hap5Δ/Δ*(TF093), and *hap43Δ/Δ*(RPC75) strains. Cells were grown in iron-replete (+Fe; FAS) or iron-deficient (−Fe; BPS) conditions, cross-linked with formaldehyde, harvested, lysed, and the chromatin extracts were prepared by sonication. Equal amounts of chromatin extracts were used for immunoprecipitation with anti-H3 K9Ac or as control anti-H3 antibodies and the immunoprecipitated DNA was analyzed by qPCR using primer pairs for *FRP1* and *ACO1* promoters or a Chr I region as a nonspecific control. The data was quantified from three biological replicates each and the error bars represent SEM (n = 3). *p*-value ≤ 0.05 (∗), ≤0.01 (∗∗), ≤0.001 (∗∗∗), and >0.05 (ns). *C* and *D*, RNA Pol II recruitment is stimulated by Sef1 and the HAP complex under iron-deficient conditions. Pol II recruitment was analyzed by ChIP assay at *C, FRP1* and *D, ACO1* promoters in the wild-type strain (WT; SN152), or the *sef1Δ/Δ* (TF015), *hap5Δ*/*Δ* (TF093), and *hap43/ΔΔ* (RPC75) mutant strains. Cells were grown in iron-deficient or iron-supplemented media for 5 h and cross-linked with formaldehyde, lysed, and chromatin extracts were prepared by sonication. Equal amounts of chromatin extracts from WT and mutant strains were used for chromatin immunoprecipitation using antibody against Pol II CTD phosphorylated on Ser5P (4H8). The enrichment of Pol II occupancy at *FRP1* or *ACO1* promoters was normalized to a nonspecific control Chr I region (ca21chr1_1573500–1574000) and plotted as fold Pol II enrichment of specific region with reference to the background. The quantification is based on at least three biological replicates and the error bars represent SEM (n = 4). *p*-value ≤ 0.05 (∗), ≤0.01 (∗∗), and >0.05 (ns). *E*, requirement of TFs for regulation of expression of *FRP1* and *ACO1* mRNAs. Total RNA was isolated from cultures grown for 5 h in BPS plus FAS (+Fe) or BPS (−Fe) and qRT-PCR was carried out with primers specific for *FRP1* and *ACO1*ORFs, and the fold change in expression in −Fe *versus* +Fe or +Fe *versus* −Fe, normalized to *sCR1* as endogenous control and plotted. *p*-value ≤ 0.05 (∗) and >0.05 (ns).

Next, we assessed the status of nucleosomal histone H3-Lys9 acetylation, a transcriptional activation-linked chromatin mark, at the two iron homeostasis gene promoters. The H3-K9Ac level, normalized to total H3, was determined at the *FRP1* promoter by ChIP assay. The data showed that *FRP1* promoter was hypoacetylated in Fe-replete medium in the WT strain, but was strongly hyperacetylated in Fe-deplete medium (Fig. 6*A*). Deletion of *SEF1*, *HAP5*, or *HAP43* reduced the hyperacetylation in Fe-deplete medium (Fig. 6*A*). At the *ACO1* promoter, the H3-K9Ac level was strongly elevated in Fe-replete medium in the WT strain (Fig. 6*B*), and this hyperacetylation was dependent on both Sef1 and Hap43, but not on Hap5 (Fig. 6*B*). In Fe-deplete medium, the *ACO1* promoter was not hyperacetylated at the H3-K9, but interestingly, *hap43*Δ/Δ and *hap5*Δ/Δ mutations led to hyperacetylation (Fig. 6*B*). Three conclusions can be drawn based on these ChIP data. First, at the *FRP1* promoter, Sef1, Hap5, and Hap43 stimulate H3K9 hyperacetylation under −Fe condition. Second, at the *ACO1* promoter, Sef1 and Hap43 promote H3K9 hyperacetylation under +Fe condition, and third, Hap5 and Hap43 repress H3K9 acetylation at the *ACO1* promoter under −Fe condition.

### RNA polymerase II recruitment during iron homeostasis transcriptional control

To examine the recruitment of Pol II to *FRP1* and *ACO1* promoters, we carried out ChIP analysis in the WT and the *hap43Δ*, *hap5Δ*, and *sef1Δ* mutant strains using an RNA pol II CTD phospho Ser5 antibody. We found that Pol II occupancy at the *FRP1* promoter is highly dependent on Sef1 and Hap5 in −Fe medium (Fig. 6*C*). Interestingly, the *hap43*Δ mutation led to elevated Pol II levels at the *FRP1* promoter compared with that in WT strain in −Fe medium (Fig. 6*C*). In +Fe medium, however, the *sef1Δ*, *hap5*Δ, and *hap43*Δ mutations did not significantly alter Pol II levels (Fig. 6*C*) at *FRP1* promoter.

At the *ACO1* promoter, we observed a high level of Pol II enrichment in +Fe medium in a manner dependent on Sef1 but not Hap5 and Hap43 (Fig. 6*D*). In contrast, no Pol II enrichment was seen at this promoter in −Fe medium. Interestingly, the *hap5Δ* and *hap43Δ* mutations, but not the *sef1Δ* mutation, led to a significant increase in Pol II occupancy compared with that in WT strain at the *ACO1* promoter in −Fe medium (Fig. 6*D*).

To correlate the H3K9 acetylation and Pol II occupancy to the transcription state of *FRP1* and *ACO1* genes, we measured mRNA levels by qRT-PCR. *FRP1* mRNA was highly induced (∼80-fold) in −Fe medium compared with +Fe medium in WT strain (Fig. 6*E*). The deletion of *SEF1*, *HAP5*, or *HAP43* genes substantially reduced *FRP1* induction in −Fe medium. The *ACO1* mRNA was strongly downregulated in the WT strain in −Fe medium compared with +Fe medium (Fig. 6*F*). Interestingly, deletion of *HAP5* or *HAP43*, but not *SEF1*, caused derepression of *ACO1* expression as the −Fe/+Fe expression ratio was ∼1.0 in the mutants compared with ∼0.04 in the WT strain indicating the repressive activity of *HAP5* and *HAP43* at the *ACO1* gene (Fig. 6*F*). Moreover, the derepression of *ACO1* expression upon *HAP5* or *HAP43* deletion is consistent with the elevated H3K9Ac level (Fig. 6*B*) and Pol II occupancy (Fig. 6*D*). Although *hap43*Δ/Δ mutation led to elevated Pol II recruitment at both *FRP1* and *ACO1* promoters under −Fe conditions (Fig. 6, *C* and *D*), only *ACO1* mRNA level is derepressed in −Fe medium suggesting a requirement for further activation of *FRP1* promoter postrecruitment of Pol II. Together, the H3-K9Ac and Pol II occupancy data along with the mRNA data indicated that while Sef1 functions as an activator of Pol II recruitment and transcription of *FRP1* in −Fe medium and that of *ACO1* in +Fe medium, the HAP trimeric complex functions as a dual regulator of Pol II recruitment and transcription, *i.e.*, as an activator at *FRP1*, and as a repressor at *ACO1* promoters.

### SAGA complex mediates iron homeostasis gene expression

**Figure 7:**
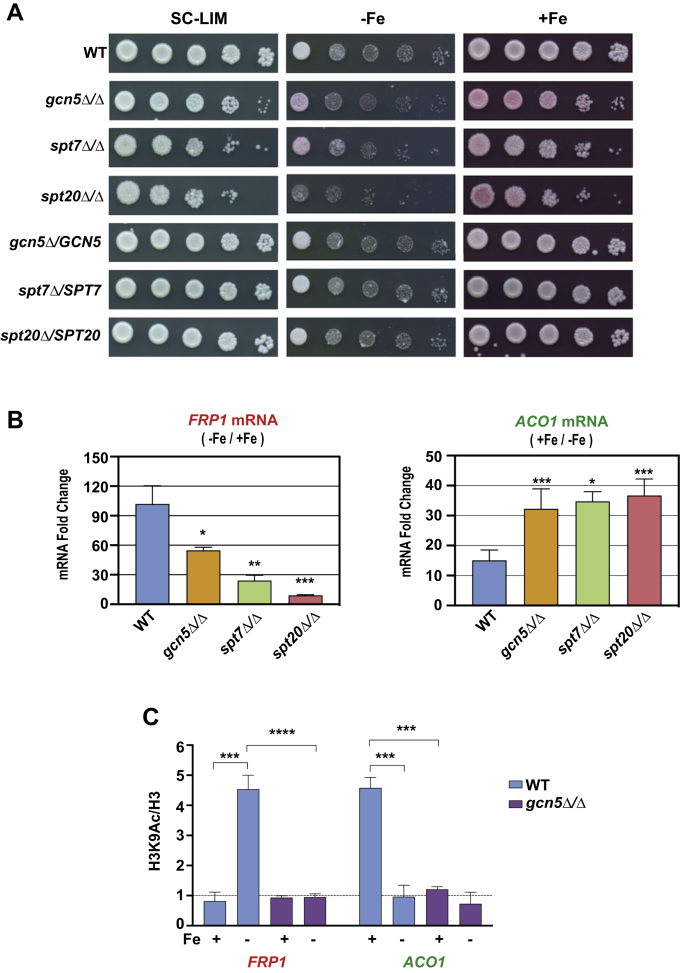
**SAGA complex is required for *C. albicans* resistance to iron deprivation stress and transcriptional regulation and histone acetylation.***A*, SAGA subunit mutants are impaired for growth in iron-depleted medium. The WT parental strain (SN152), or the *gcn5Δ/Δ* (KNC642), *spt7Δ/Δ* (KNC652), and *spt20Δ/Δ* (KNC654) mutant strains and the isogenic complemented strains bearing an integrated copy of the wild-type *GCN5* (PRI1), *SPT7* (PRI2), and *SPT20* (PRI3) genes were grown in SC-LIM medium and 10-fold serial dilutions were spotted on SC-LIM alone or with either 100 μM BPS (−Fe) or 100 μM each FAS and BPS (+Fe) and growth monitored. *B*, SAGA complex has a dual role for expression of genes involved in iron homeostasis. The WT parental strain and the different SAGA mutants were grown in BPS (−Fe) or BPS plus FAS (+Fe) media, total RNA isolated, and mRNA levels were assayed by qRT-PCR using primers specific for *FRP1* and *ACO1* mRNAs or for *sCR1* RNA as control, quantified by relative C_*T*_ method, and the data was plotted as fold change (−Fe/+Fe). The error bars represent SEM (n = 3). *C*, Histone H3-Lys9 acetylation of iron homeostasis gene promoters is mediated by Gcn5. *C. albicans* strains were grown in −Fe or +Fe media, chromatin extract was prepared and immunoprecipitated with H3 Lys9-Ac antibody or with control pan anti-histone H3 antibody, the input DNA and IP-DNA were purified and quantified by qRT-PCR using primers for *ACO1* and *FRP1* promoters and a control region from chromosome 1 (ca21chr1_1573500–1574000). The ChIP enrichment was plotted as a ratio of H3-Lys9Ac normalized to total H3 level, and the error bars represent SEM (n = 3). *p*-value ≤ 0.05 (∗), ≤0.01 (∗∗), ≤0.001 (∗∗∗), ≤0.0001 (∗∗∗∗), and >0.05 (ns).

The Gcn5-containing SAGA complex is the major histone acetyl transferase that mediates H3K9 acetylation in yeast (26, 27). Therefore, to test if the SAGA complex is required for iron homeostasis gene regulation, we constructed three *C. albicans* strains bearing deletion mutations in SAGA subunits, namely *GCN5* encoding the CaKAT2 histone acetyl transferase and two key SAGA-specific subunits *SPT7* and *SPT20*. The mutants and the control WT strains were tested for growth in −Fe condition (+BPS plates). Compared with the WT parental strain, the *gcn5Δ*, *spt7Δ*, and *spt20Δ* null mutants were impaired for growth in −Fe plate (Fig. 7*A*). Of the three, the *spt20Δ* mutant showed the strongest growth defect as compared with the *gcn5Δ* and *spt7Δ* mutants. The three mutants also showed slow-growth phenotype in the control SC-LIM plate, and thus the BPS-sensitive growth defect appears to be less pronounced in BPS-containing plate (−Fe; Fig. 7*A*). However, iron supplementation to the BPS plate rescued most of the growth defect of the mutants (+Fe; Fig. 7*A*). Also, reintroduction of a WT copy of *GCN5*, *SPT7*, and *SPT20* genes in the respective mutants complemented the growth defects in −Fe as well as in the control plate (Fig. 7*A*) indicating that the three SAGA genes are required for *C. albicans* response to iron deprivation.

Next, we analyzed if the SAGA subunit genes are required for expression of the iron homeostasis genes *FRP1* and *ACO1*. The expression of *FRP1* mRNA was induced ∼100-fold in −Fe (+BPS) medium compared with +Fe medium (+FAS) in the WT strain (Fig. 7*B*). The *FRP1* mRNA induction, however, was significantly reduced in all three SAGA mutants, namely *gcn5Δ/Δ*, *spt7Δ/Δ*, and *spt20Δ/Δ* (Fig. 7*B*), with the reduction between ∼2.0-fold in *gcn5Δ/Δ* and ∼10.0-fold in the *spt20Δ/Δ* mutant, suggesting that mutations in the structural components of SAGA had a major impact possibly due to the *spt7Δ/Δ* and *spt20Δ/Δ* mutations substantially affecting the integrity of the SAGA complex as reported previously for the budding yeast SAGA (28, 29, 30). The *ACO1* mRNA expression had a fold change (plotted as +Fe/−Fe ratio) of ∼15.0, indicating that *ACO1* expression is repressed in −Fe medium compared with +Fe medium in the WT strain (Fig. 7*B*). In the *gcn5Δ*, *spt7Δ*, and *spt20Δ* mutants, unexpectedly, the *ACO1* mRNA expression was further elevated in +Fe medium (Fig. 7*B*) indicative of a repressive function for SAGA at this promoter under Fe-replete conditions. In any case, these data provided evidence that the SAGA complex is a critical effector of iron homeostasis gene regulation in *C. albicans*.

To further establish a direct role for acetylation by Gcn5, we assessed nucleosomal histone acetylation at the two iron homeostasis gene promoters in WT and *gcn5Δ* mutant strains. ChIP assays were carried out from chromatin extracts prepared from WT and *gcn5Δ* mutant strains cultured under iron-deplete or iron-replete conditions, using either histone H3-K9 acetyl antibody or the total H3 antibody, and *FRP1* and *ACO1* promoters were probed using qPCR. The results depict that histone H3-K9 is hyperacetylated at the *FRP1* promoter in −Fe medium compared with +Fe in WT cells (Fig. 7*C*). However, in the *gcn5*Δ mutant strain, the hyperacetylation was completely lost indicating that Gcn5 is the critical histone acetyl transferase under iron-deficient conditions (Fig. 7*C*). At the *ACO1* promoter, however, we found hyperacetylation in +Fe consistent with a high level of *ACO1* mRNA expression in +Fe compared with −Fe medium (Fig. 7*C*). The *gcn5*Δ abrogated H3K9 acetylation at *ACO1* promoter in +Fe medium (Fig. 7*C*), demonstrating that SAGA-mediated acetylation is also critical for *ACO1* expression in +Fe medium.

## Discussion

Fungal pathogens encounter vastly differing iron levels in human host environments. Accordingly, *C. albicans* and other fungal pathogens maintain iron homeostasis by modulating their intracellular iron pool through multiple iron acquisition pathways and by conserving iron pools by downregulating iron-requiring proteins. The iron homeostasis gene regulation is controlled by multiple transcriptional regulators in *C. albicans* and other fungi (4, 6, 9). Previous work had shown that transcriptional regulators Sef1, HAP complex, Hap43, and Sfu1 control iron homeostasis gene regulation by promoter binding (10, 11, 13, 14, 15). There were also indications that this control could involve interlinked expression of the transcriptional regulators, but the extent of connectivity and the mechanism of their control were not understood. Therefore, in this work we rigorously examined the mutual transcriptional control of the HAP complex, Sef1, and the Hap43 under iron-deplete and iron-replete conditions. Moreover, how these iron-responsive regulators exert transcriptional control of iron homeostasis genes also was not understood.

We first probed the regulatory interactions within the HAP transcriptional regulators and found that the Hap2 and Hap5 formed a heterodimeric complex in cell extracts independent of the iron status in cells. However, the Hap2-Hap5 recruitment to *FRP1* and *ACO1* promoters was intimately linked to forming a heterotrimeric complex with the Hap3 subunit under iron starvation conditions. The expression of Hap3 in turn is controlled by Hap43 and Sef1 transcriptional regulators under iron starvation conditions, although the *HAP3* homolog NFY-B is not known to be transcriptionally controlled in metazoans. However, *C. albicans* genome encodes an additional *HAP3*-like gene *HAP31* (14, 31, 32), whose expression is not iron-regulated, and therefore, it remains to be tested if the Hap2-Hap5 interaction observed by us in iron-replete conditions could involve the Hap31 protein.

Next, we explored mutual regulation of HAP complex subunits and the Hap43 regulator. Previous studies showed that Hap43 is required for transcriptional induction of iron uptake genes including *FRP1* (11, 14), but Hap43 was recruited to the promoter of certain iron uptake genes (11). Therefore, to determine the interdependency, if any, of the HAP trimeric complex and Hap43 for promoter recruitment, we first carried out ChIP-qPCR validations for iron-deprivation-induced *FRP1*, *FTR1*, *SIT1*, and iron-deprivation-repressed *ACO1* and *CYC1* promoters (Fig. 3). Whereas Hap2, Hap3, and Hap5 were recruited to both classes of promoters, Hap43 was recruited to only the repressed gene promoters. By conducting the ChIP assays in the different HAP mutant strains, we found that Hap5 recruitment to *FRP1* and *ACO1* is dependent on Hap2 as well as Hap43 (Fig. 4, *A* and *B*), indicating that although Hap43 is not recruited to the *FRP1* promoter, the HAP trimeric complex recruitment is Hap43-dependent. Given that Hap43 is bound to multiple iron uptake genes that are induced under −Fe conditions, we would not be able to rule out if the Hap43 cross-linking efficiency at the *FRP1* promoter is weaker than that at other promoters identified in the Chip-ChIP study. Besides, Hap3 is essential for Hap2-Hap5 interaction in cell extracts (Fig. 2, *C–F*) indicating that the efficient assembly of the trimeric complex is also dependent on intersubunit interactions within the HAP complex. Thus, it is conceivable that the HAP complex assembly in *C. albicans* is a two-step process similar to that in *Aspergillus* spp. and mammals (14, 33, 34, 35, 36). Together these results highlight a mutual dependence between the subunits of the HAP complex and between Hap43 and the HAP complex for promoter recruitment.

**Figure 8:**
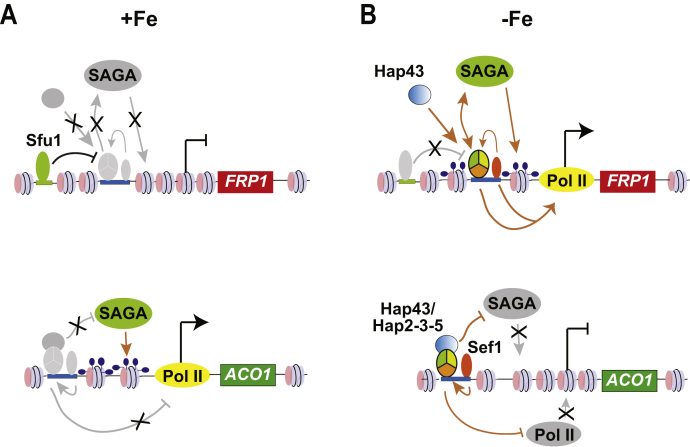
**Summary of transcriptional regulators and histone acetylation dynamics at *FRP1* and *ACO1* promoters under iron-deplete and ion-replete conditions.** Both Sef1 and Hap43 activate *HAP3* and *HAP2* transcription thereby leading to a productive Hap5/Hap2/Hap3 trimeric complex that is recruited to the *FRP1* and *ACO1* promoters. Sef1 is required for recruitment of HAP trimeric complex to *FRP1* promoter, leading to histone H3-Lys 9 acetylation by SAGA, and Pol II recruitment to induce *FRP1* expression. Although Sef1 and the HAP complex are recruited to *ACO1* promoter, this promoter is hypoacetylated at H3-K9 under Fe-deplete conditions. While Hap43 is not recruited to *FRP1*, it is efficiently recruited to the promoters of *ACO1* and to that of the GATA factor *SFU1* under Fe-deplete conditions. Recruitment of Cap2-HAP complex to *ACO1* promoter led to inhibition of SAGA activity and hypoacetylated nucleosomes and impaired Pol II recruitment at this promoter. Under Fe-replete conditions, expression of Sef1, Hap43, and Hap3 expression is downregulated and consequently their recruitment to *FRP1* is lost. Moreover, under Fe-replete conditions, the Sfu1 binds to the promoters of *FRP1* and *SEF1* to repress transcription. *Solid arrows* indicate direct regulation, the *double-headed arrows* indicate recruitment likely through protein–protein interactions, and *gray arrows* indicate loss of regulation/repression. The *black, blunt arrow* from Sfu1 indicate data from previous studies, and the *black arrow* on the promoter is indicative of transcription start site, and the rest of the *brown arrows* represent results presented in this study.

It was previously reported that ∼7% of the iron-responsive gene promoters are bound by both Hap43 and Sef1 indicating a synergy between the two regulators. Therefore, to test the interdependency of Sef1 and the HAP complex for their recruitment to *FRP1* promoter, we mutated the binding site for the HAP complex (CCAAT box) and the presumptive Sef1-binding site (CGG motif) on the *FRP1* promoter and found that while the HAP complex recruitment was dependent on Sef1, the recruitment of Sef1 itself was independent of the HAP complex indicating that Sef1 acts upstream to the HAP complex at the *FRP1* promoter. As Sef1 has been shown to be recruited to the *HAP3* promoter and induce transcription (11), Sef1 likely promotes the assembly and recruitment of the HAP complex to the *FRP1* promoter (Fig. 8*B*). Furthermore, the Sef1 bound at the *ACO1* promoter could also potentially promote the recruitment of the HAP trimeric complex and Hap43 to the *ACO1* promoter (Fig. 8*B*).

A previous nucleosome mapping study identified a reduced nucleosome occupancy at the *FRP1* promoter under Fe-deplete conditions (37). At the *ACO1* promoter, however, the nucleosome occupancy was high under Fe-deplete compared with Fe-replete conditions (37). These differential nucleosome occupancy data are consistent with the transcriptional states of the two promoters under Fe-deplete and Fe-replete conditions (Fig. 8, *A* and *B*) indicating a chromatin-mediated control of iron homeostasis. Our results established that the SAGA complex subunits encoded by *GCN5*, *SPT7*, and *SPT20* are required for cellular growth under Fe-depleted conditions and for optimal regulation of *FRP1* and *ACO1* promoters under Fe-deplete and Fe-replete conditions respectively (Fig. 7, *A* and *B*). Although *FRP1* expression was impaired in all three SAGA mutants tested, the *spt7Δ* and *spt20Δ* mutations had a greater impact on gene expression as compared with *gcn5Δ* (Fig. 7*B*), with *spt20Δ* being most debilitating. There are compelling previous reports indicating such differential SAGA subunit effects. Proteomics studies of ySAGA (28, 29, 30) showed that loss of *spt7Δ* or *spt20Δ* caused a substantial impact on the integrity of SAGA complex in budding yeast. The genome-wide transcriptome study (38) on the different ySAGA subunit deletions showed that *spt7Δ* and *spt20Δ* impacted a larger number of genes at global level as compared with *gcn5Δ*. Additionally, the single subunits deletions (*gcn5Δ or spt7Δ* or *spt20Δ*) have less effect on mRNA levels than the double deletions (*gcn5Δ spt3Δ*). Moreover, deletion of subunits of a particular SAGA module showed similar expression profiles, whereas deletion of subunits of different SAGA modules impacted different subset of genes (39). The differential effect of the *SPT7* and *SPT20* subunits was also observed in the recruitment of RNAPII in *spt7Δ* and *spt20Δ* (40). So again, based on the different effects of *spt7Δ*, *spt20Δ*, and *gcn5Δ* on *FRP1* and *ACO1* expression, there seems to be different requirements for SAGA in activation and repression, also indicating that multiple SAGA functions could be invoked in gene expression control of iron homeostasis.

The Sef1, HAP complex, and Hap43 promote H3-K9 hyperacetylation of the *FRP1* promoter under Fe-deplete conditions (Fig. 6*A*). Furthermore, Sef1 and the HAP complex are also required for Pol II recruitment at the *FRP1* promoter under Fe-deplete conditions (Fig. 6*C*). At the *ACO1* promoter, however, Hap5 and Hap43, but not Sef1, are required to maintain this promoter in a hypoacetylated state under Fe-deplete conditions (Fig. 6*B*), consistent with the repressive function of the Hap43-HAP complex at the *ACO1* promoter. Consistent with the transcriptional repressive function of the Hap43-HAP complex at *ACO1*, Pol II recruitment is greatly stimulated upon *hap5Δ/Δ* and *hap43Δ/Δ* deletion under Fe-deplete conditions (Fig. 6*D*). Furthermore, we are surprised that the *hap43Δ/Δ* mutation led to a greater than WT level of Pol II recruitment at the *FRP1* promoter (Fig. 6*C*); but the reduced *FRP1* mRNA level in the *hap43*Δ/Δ mutant perhaps suggests that Pol II recruitment is not sufficient to activate the *FRP1* promoter under Fe-deplete conditions indicating a postrecruitment control of Pol II activation for *FRP1* transcription. We posit that the HAP complex could be minimally sufficient to directly stimulate H3-K9 acetylation upon *FRP1* promoter binding, and Sef1 could just mediate HAP complex recruitment. In this context, other transcriptional activators stimulate H3 acetylation by SAGA recruitment through activation domain interactions (41, 42, 43, 44, 45, 46), although an activation domain is yet to be identified in either Sef1 or the HAP complex.

Based on the data presented in this study together with previous studies, a model emerges wherein iron deprivation elicited the transcriptional induction of *SEF1* and *HAP43* mRNAs as well as the repression of *SFU1* mRNA expression. Sef1 and Hap43 in turn upregulated the transcription of *HAP3* and *HAP2* under iron-deplete conditions leading to a functional HAP trimeric complex. Under +Fe conditions, however, *SEF1* and *HAP43* transcription is repressed. The GATA factor Sfu1 is a negative regulator of iron uptake genes (10) and is bound to *FRP1* and other iron-uptake promoters under iron-replete conditions and repress transcription (11). Moreover, as the HAP complex and Sef1 are absent under +Fe, the *FRP1* promoter remains deacetylated (Fig. 8*A*). Paradoxically, the loss of HAP complex in +Fe led to a hyperacetylated state of *ACO1* promoter (Fig. 8*B*). It is conceivable that the *ACO1* hyperacetylation in +Fe is likely due to SAGA activity recruited by Hap43, as the deletion of *HAP43* (Fig. 6*B*) or *GCN5* (Fig. 7*C*) impaired H3K9Ac at *ACO1* under +Fe condition.

Under −Fe conditions, a functional Hap2-Hap3-Hap5 trimeric complex and Sef1 are recruited to *FRP1*, while the HAP trimeric complex, Hap43, and Sef1 are recruited to the promoter of *ACO1* (Fig. 8*B*). The recruitment of the trimeric HAP complex and Sef1 stimulates SAGA activity and Pol II recruitment at *FRP1* leading to transcriptional activation of *FRP1* (Fig. 8*B*). Although Hap43 is not occupied at *FRP1*, Hap43 is required for *HAP3* expression and thereby stimulates HAP recruitment to *FRP1*. The Sef1 regulator induces the expression of *HAP43* as well as *HAP3* under −Fe conditions and thus also stimulates HAP complex assembly at the *FRP1* and *ACO1* promoters (Fig. 8, *A* and *B*). In contrast, recruitment of trimeric HAP complex and Hap43 to the *ACO1* promoter leads to inhibition of SAGA activity and the consequent loss of *ACO1* expression in −Fe medium (Fig. 8*B*). Thus, the Hap2-Hap3-Hap5 trimeric complex and Hap43 have dual roles in regulation of iron homeostasis by transcriptional activation at *FRP1* and repression of *ACO1* (14).

Together the data presented here indicated that *C. albicans* has evolved a fail-safe mechanism for iron homeostasis transcriptional control by incorporating the HAP complex, Sef1, and Hap43, together with SAGA-mediated chromatin modification, as subswitches in turning on the transcriptional control in this highly pervasive human fungal pathogen. The SAGA-mediated control of iron homeostasis gene expression also provides a highly tunable system for a rapid response of *C. albicans* to changing iron milieu in the host. It would be fascinating to explore what elements of this intricate control are also operative in the vastly differing iron levels in mammalian tissues that are infected by *C. albicans*. Given that the genes encoding the HAP complex and Sef1 and the SAGA subunit genes *ADA2*, *GCN5, SPT20* and *SPT3* are required for *C. albicans* virulence (47, 48, 49, 50), a chromatin level epigenetic control of transcription seems a pivotal means of iron homeostasis regulation.

## Experimental procedures

### Strains and growth conditions

*C. albicans* strains were cultured in YPD or in synthetic complete limited iron medium (SCLIM) as described previously (14). Iron-depleted media used was either SC-LIM or YPD with 100 μM BPS or 200 μM BPS respectively. For iron-replete media, 100 μM FAS (SC-LIM) or 200 μM FAS (YPD) was supplemented to deplete media. *C. albicans* cells were cultured in either SC-LIM or YPD media and grown for 14–16 h at 30 °C and diluted to an initial OD of 0.25 (WT) or 0.5 (mutants) and grown for 5 h and harvested, and cell pellets were used immediately or snap frozen in liquid nitrogen and stored at −80 °C till use.

### Construction of strains and plasmids

The *C. albicans* strains and plasmids used in this study are listed in the Table S1 and S2. All strains in this study are derived from *C. albicans* parental strain SN152 and SN95 (37). Oligonucleotides used in this study are listed under Table S3.

Details regarding construction of plasmids and *C. albicans* strains are available on request. Briefly, genes encoding the HAP complex subunits Hap2, Hap3, and Sef1 were genomically TAP-tagged in *C. albicans* strain SN152 using the plasmids Ip21 and Ip22 (25) as DNA templates and ONC645/646, ONC649/650, ONC637/638 respectively as primers and the tagging cassettes were amplified by PCR using Phusion HF DNA polymerase. Correct integration of the tagging cassettes in *C. albicans* strains KNC398, KNC410, and KNC379 was confirmed by PCR, and protein expression was confirmed by western blotting using rabbit polyclonal anti-TAP antibody (Thermo Fisher). The Hap5-TAP strain RPY453 was described previously (14). The mutant strains KNC430, KNC453, and RPY471 were constructed by deleting *sef1Δ*, *hap2Δ*, and *hap43Δ* in strain RPY453. The mutant strain KNC402 was constructed by TAP tagging the *SEF1* gene in strain RPY431. The various deletions were confirmed by PCR and the expression of TAP-tagged proteins by western blotting.

The strains KNC692 (WT) and KNC693 (*hap2Δ*) bearing Hap5-TAP and Hap3-FLAG were constructed by integration of the His_6_-FLAG_3_ cassette from plasmid pSH26-5 (14) into the *HAP3* locus in strains RPY453 and KNC453 respectively. The strain KNC553 bearing Hap2TAP and Hap5-FLAG was constructed by integration of the His_6_-FLAG_3_ cassette from plasmid pSH26-5 into the *HAP5* locus in strain KNC398. The strain KNC558 was constructed in multiple steps as follows. First, the *HAP5* locus was FLAG-tagged in strain RPY431, and subsequently, the *HAP2* locus was TAP-tagged to obtain strain KNC558.

To construct *gcn5Δ/Δ*, *spt7Δ/Δ*, and *spt20Δ/Δ* deletion strains, we carried out sequential deletion of both alleles using the *HIS1-ARG4-HIS1* (HAH) deletion cassette from plasmid pHAH1 (51), using primers for *GCN5* (ONC601/ONC115 and ONC601/ONC114), *SPT7* (ONC602/ONC115 and ONC603/ONC114), or *SPT20* (ONC605/ONC115 and ONC606/ONC114) and amplified the respective up-split and down-split fragments, combined and transformed into *C. albicans* strain SN95 and selected for Arg^+^ colonies. The correct integrations were confirmed using PCR, and the resultant heterozygous strains KNC641, KNC651, and KNC653 were grown nonselectively in YPD and Arg^+^ His^+^ colonies representing homozygous deletion strains KNC642, KNC652, and KNC654 were selected and confirmed by PCR.

For reintegration of *GCN5*, *SPT7*, and *SPT20* into the homozygous deletion strains KNC642, KNC652, and KNC654, plasmid pNIM1 was sequentially digested with BglII/MluI to obtain nourseothricin resistance marker *CaSAT1* while plasmid CIp10 was digested with NotI/XbaI and end filled with Klenow. Then linearized plasmid pCIp10 and insert CaSAT1 was ligated and transformed to electrocompetent bacterial cells DH10B to obtain plasmid pCIp10-SAT1(pPI1). Then, genes were amplified along with 1000 bp upstream and 500 bp downstream of ORF sequences using primer pairs ONC1156-ONC1157 (*GCN5*), ONC1154-ONC1155 (*SPT7*), and ONC1152-ONC1153 (*SPT20*) respectively. The amplified product was digested with BamHI (for *SPT7* and *SPT20*) and SacI (for *GCN5*) and cloned into plasmid pPI1 digested with the respective enzymes and plasmids pPI2 (*GCN5*), pPI3 (*SPT7*), and pPI4 (*SPT20*) were obtained.

The plasmids pPI2, pPI3, and pPI4 were linearized with PmeI, BstEII, and Afl II respectively and transformed into KNC642, KNC652, and KNC654 strains, and positive transformants were confirmed by PCR using ORF specific primer pairs ONC661/ONC662, ONC663/ONC664, ONC708/ONC709 and obtained PRI1 (*gcn5Δ/GCN5*), PRI2 (*spt7Δ*/*SPT7*), and PRI3 (*spt20Δ/SPT20*) respectively.

Strains for β-Gal assays were constructed as follows. The *P*_*ACT1*::_*lacZ*-*ACT1*t insert from plasmid pAU36 (52) was subcloned into CIp10 (53) to construct pNA36. A 1kb *HAP43* promoter region was amplified from SC5314 strain genomic DNA and cloned as a KpnI-SalI fragment into the KpnI-XhoI digested pNA36 to replace *P*_*ACT1*_ and obtained plasmid pNA40. The plasmid pNA40 was linearized with StuI and transformed into SN152, RPC75, and TF015 (*sef1Δ*) by electroporation to integrate the reporter plasmid at the *RPS1* locus and obtained KNC332, KNC340, and KNC348. The correct integration was confirmed by PCR.

The strains bearing WT and mutant *FRP1* promoters were constructed in several steps. First, the WT *FRP1* coding sequence along with 500 bp upstream region was deleted in strains RPY453 and KNC379 to obtain strains KNC467 and KNC481 respectively. The homozygous *frp1Δ* deletion was confirmed by PCR. Next, a DNA fragment containing the *FRP1* coding sequence flanked by 1kb upstream region and 300 bp downstream region was amplified as a KpnI-SacI fragment using ONC630-ONC631 and cloned into pLitmus28 to obtain pNA51. The WT *FRP1* fragment was then subcloned into pSN69 (54) and obtained plasmid pNA61, which was integrated at the native *FRP1* locus in strain KNC467 and KNC481 and obtained strains KNC479 and KNC486 respectively. The correct integration was confirmed by PCR.

To construct point mutations in the HAP complex binding site (CCAAT motif) in the *FRP1* promoter, the mutagenic oligonucleotide pair ONC901-ONC902 was designed using the Quick Change Primer Design tool (Agilent Technologies) and the CCAAT motif (−134 with reference to ATG) was mutated to TGCGC as reported previously (13). The plasmid pNA61 was used as a template along with the mutagenic primers and amplified using the Quick-Change II XL Site-Directed Mutagenesis Kit and obtained plasmid pNA68. Similarly, the putative Sef1-binding site (11) was mutated using the mutagenic oligonucleotides ONC929-ONC930 and ONC931-ONC932 and obtained plasmid pNA76. The WT and the two mutant insert sequences in the plasmid clones were verified by Sanger sequencing. Next, the plasmids pNA68 and pNA76 were linearized with Bpu101, gel purified, and integrated at the chromosomal *frp1* locus in strains KNC467 and KNC481 and obtained strains KNC494, KNC498, KNC505, and KNC509. The correct integrations were confirmed by PCR.

### β-galactosidase assay

Briefly, three independent transformants of each strain were grown overnight in SC-LIM and then diluted to 30 ml SC-LIM plus 100 μM BPS (−Fe) at a starting density of 0.3 OD_600_ and 0.5 OD_600_ for WT and the mutant strains (*hap43Δ/Δ* and *sef1Δ/Δ*), respectively. For iron-replete conditions (+Fe; SC-LIM plus 100 μM BPS and 100 μM FAS), cells were diluted to a starting density of 0.25 OD_600_ for all the strains and grown for 5 h till ∼1.0 OD. Cells were harvested and cell pellets washed with Z-buffer (55), resuspended in 200 μl Z-buffer with 2 mM PMSF, and vortexed for 2 min in cold room using a Braun homogenizer. Supernatants were collected and protein extracts were cleared by centrifugation at 13,000 rpm at 4 °C for 15 min. The total protein concentration in the cell lysates was determined by Bradford assay using Softmax Pro 2.6 program in SpectraMax 340 microplate reader (Molecular Devices). The β-galactosidase kinetic assay was performed at 37 °C in 96-well microplates in SpectraMax 340 microplate reader with ONPG as substrate (0.75 mg/ml), absorbance monitored, and the β-galactosidase specific activity was calculated as described previously (56) and plotted using GraphPad Prism 6. Statistical analysis of Hap43 protein level and the β-galactosidase activity was performed by comparing expression in −Fe compared with +Fe and statistical significance determined by Student's *t*-test.

### Whole-cell extract preparation, coimmunoprecipitation, and Western blotting

The cell pellets were resuspended in chilled Winston Buffer (40 mM HEPES-NaOH pH 7.5, 350 mM NaCl, 10% glycerol, 0.1% Tween 20 (57) with added protease inhibitors (2.5 μg/ml Aprotinin, 2 mM Benzamidine, 1 mM Dithiothreitol, 2 μg/ml Leupeptin, 2 μg/ml Pepstatin, 100 μM PMSF, 10 μg/ml TPCK, 10 μg/ml TLCK), and vortexed in presence of glass beads. The lysates were cleared by centrifugation at 13,000 rpm for 15 min at 4 °C.

Five milligrams of whole-cell extracts was used for immunoprecipitation with IgG-Sepharose 6 Fast Flow beads (GE healthcare) for 4 h at 4 °C, washed with Winston buffer, followed by TEV buffer. The beads were then incubated with AcTEV protease (Invitrogen) overnight at 4 °C. The eluate was collected and 20% of total eluate (IP) and 100 μg whole-cell extracts (Input) were separated on 10% SDS-PAGE gel, transferred onto a Protran nitrocellulose membrane (GE healthcare) and probed with anti-FLAG, anti-TAP oranti-Glucose-6-P-dehydrogenase antibodies detected with ECL-Prime Western blot detection reagent (GE Healthcare) and exposed to X-ray film.

### RNA analysis

Total RNA was isolated from *C. albicans* cells and cDNA was synthesized essentially as described before (14). Real-time qRT-PCR was carried out in Applied Biosystems Fast 7500 Real-time PCR system using gene-specific primers and differential expression was calculated by the comparative *C*_*T*_ method (58). The *SCR1* RNA, an RNA polymerase III transcript was used as endogenous control (14).

### Chromatin immunoprecipitation (ChIP) assay

ChIP assays were conducted essentially as described before (14). Briefly, all strains were precultured overnight in SC-LIM medium, grown in fresh SC-LIM medium with 100 μM BPS alone or with BPS and 100 μM FAS for 5 h at 30 °C to an OD_600_ of 0.8–1.0, cross-linked with 1% (v/v) formaldehyde, quenched with 125 mM glycine, cells harvested, and chromatin extracts prepared by shearing in Bioruptor (model UCD 300, Diagenode). For chromatin immunoprecipitation of TAP-tagged proteins, the sheared chromatin extracts equivalent to ∼25OD_600_ cells from control and experimental strains were immunoprecipitated with about 25 μl of IgG-Sepharose 6 Fast Flow (pre-blocked with 0.2 mg/ml sheared Calf thymus DNA and 0.1% (w/v) BSA) for 3 h at 4 °C. For Hap43 ChIP, 2 μl affinity-purified antibody (10) was bound to 30 μl beads and immunoprecipitated for 4 h at 4 °C. The chromatin immunoprecipitations were conducted using chromatin extract from ∼35 OD600 equivalent cells and 2 μg of each antibody prebound to Dynabeads Protein G. The following antibodies were used in ChIP: anti-total H3, anti-H3-K9Ac, and RNA pol II CTD phospho Ser5 antibody (4H8, Abcam).

Immunoprecipitation was carried out for 9 h at 4 °C, washed, and eluted in 25 μl 0.1 × TE. The input DNA and immunoprecipitated DNA were purified, diluted 1:10,000 (Input DNA) and 1:5 (IP DNA), and probed by quantitative real-time PCR for specific regions of interest as well as for the control nonspecific region (ca21chr1_1573500–1574000). For all chromatin immunoprecipitations, enrichment was calculated for target regions and for the control nonspecific region (ca21chr1_1573500–1574000) and specific enrichment calculated with respect to input total chromatin. For the TAP chromatin immunoprecipitations, the fold enrichment was determined by further normalizing the background amount of immunoprecipitation obtained in mock immunoprecipitation conducted using chromatin extracts from untagged strain. Similarly, for Hap43 chromatin immunoprecipitations, the specific enrichment was calculated by further normalizing the background amount of immunoprecipitation obtained in the *hap43*Δ/Δ mutant chromatin immunoprecipitation.

## Statistical analysis

The statistical significance of quantitative data was determined by Student's *t*-test in Graph Pad Prism 6, and a *p*-value ≤ 0.05 was considered statistically significant. The *p*-values are indicated as ∗ (*p* ≤ 0.05), ∗∗ (*p* ≤ 0.01), ∗∗∗ (*p* ≤ 0.001), ∗∗∗∗ (*p* ≤ 0.0001), and ns (*p* > 0.05).

## Data availability

All data reported here are contained within the article or as supporting information.

## Supporting information

This article contains supporting information (11, 13, 14, 19, 25, 51, 52, 53, 54, 55, 56, 57, 58, 59, 60, 61).

## Conflicts of interest

The authors declare that they have no conflicts of interest with the contents of this article.

## References

[1] Mayer F.L., Wilson D., Hube B. (2013). *Candida albicans* pathogenicity mechanisms. Virulence.

[2] Berman J., Sudbery P.E. (2002). *Candida albicans*: A molecular revolution built on lessons from budding yeast. Nat. Rev. Genet..

[3] Parr R.M., Taylor D.M. (1964). The concentrations of cobalt, copper, iron and zinc in some normal human tissues as determined by neutron-activation analysis. Biochem. J..

[4] Srivastav M.K., Nair R., Natarajan K., Prasad R. (2017). Iron acquisition in the pathobiology of *Candida albicans*. *Candida albicans*: Cellular and Molecular Biology.

[5] Almeida R.S., Wilson D., Hube B. (2009). *Candida albicans* iron acquisition within the host. FEMS Yeast Res..

[6] Gupta M., Outten C.E. (2020). Iron–sulfur cluster signaling: The common thread in fungal iron regulation. Curr. Opin. Chem. Biol..

[7] Mao Y., Chen C. (2019). The hap complex in yeasts: Structure, assembly mode, and gene regulation. Front. Microbiol..

[8] Blankenship J.R., Mitchell A.P. (2011). *Candida albicans* adds more weight to iron regulation. Cell Host Microbe.

[9] Noble S.M. (2013). *Candida albicans* specializations for iron homeostasis: From commensalism to virulence. Curr. Opin. Microbiol..

[10] Lan C.Y., Rodarte G., Murillo L.A., Jones T., Davis R.W., Dungan J., Newport G., Agabian N. (2004). Regulatory networks affected by iron availability in *Candida albicans*. Mol. Microbiol..

[11] Chen C., Pande K., French S.D., Tuch B.B., Noble S.M. (2011). An iron homeostasis regulatory circuit with reciprocal roles in *Candida albicans* commensalism and pathogenesis. Cell Host Microbe.

[12] Hameed S., Prasad T., Banerjee D., Chandra A., Mukhopadhyay C.K., Goswami S.K., Lattif A.A., Chandra J., Mukherjee P.K., Ghannoum M.A., Prasad R. (2008). Iron deprivation induces EFG1-mediated hyphal development in *Candida albicans* without affecting biofilm formation. FEMS Yeast Res..

[13] Baek Y.U., Li M., Davis D.A. (2008). *Candida albicans* ferric reductases are differentially regulated in response to distinct forms of iron limitation by the Rim101 and CBF transcription factors. Eukaryot. Cell.

[14] Singh R.P., Prasad H.K., Sinha I., Agarwal N., Natarajan K. (2011). Cap2-HAP complex is a critical transcriptional regulator that has dual but contrasting roles in regulation of iron homeostasis in *Candida albicans*. J. Biol. Chem..

[15] Hsu P.C., Yang C.Y., Lan C.Y. (2011). *Candida albicans* Hap43 is a repressor induced under low-iron conditions and is essential for iron-responsive transcriptional regulation and virulence. Eukaryot. Cell.

[16] Johnson D.C., Cano K.E., Kroger E.C., McNabb D.S. (2005). Novel regulatory function for the CCAAT-binding factor in *Candida albicans*. Eukaryot. Cell.

[17] Srivastav M.K., Agarwal N., Natarajan K. (2018). Multiple evolutionarily conserved domains of Cap2 are required for promoter recruitment and iron homeostasis gene regulation. mSphere.

[18] Skrahina V., Brock M., Hube B., Brunke S. (2017). Candida albicans Hap43 domains are required under iron starvation but not excess. Front. Microbiol..

[19] Homann O.R., Dea J., Noble S.M., Johnson A.D. (2009). A phenotypic profile of the *Candida albicans* regulatory network. PLoS Genet..

[20] Chen C., Noble S.M. (2012). Post-transcriptional regulation of the Sef1 transcription factor controls the virulence of Candida albicans in its mammalian host. PLoS Pathog..

[21] Li B., Carey M., Workman J.L. (2007). The role of chromatin during transcription. Cell.

[22] Lee K.K., Workman J.L. (2007). Histone acetyltransferase complexes: One size doesn't fit all. Nat. Rev. Mol. Cell Biol..

[23] Spedale G., Timmers H.T., Pijnappel W.W. (2012). ATAC-king the complexity of SAGA during evolution. Genes Dev..

[24] Helmlinger D., Tora L. (2017). Sharing the SAGA. Trends Biochem. Sci..

[25] Sinha I., Kumar S., Poonia P., Sawhney S., Natarajan K. (2017). Functional specialization of two paralogous TAF12 variants by their selective association with SAGA and TFIID transcriptional regulatory complexes. J. Biol. Chem..

[26] Grant P.A., Winston F., Berger S.L. (2021). The biochemical and genetic discovery of the SAGA complex. Biochim. Biophys. Acta Gene Regul. Mech..

[27] Soffers J.H.M., Workman J.L. (2020). The SAGA chromatin-modifying complex: The sum of its parts is greater than the whole. Genes Dev..

[28] Lee K.K., Sardiu M.E., Swanson S.K., Gilmore J.M., Torok M., Grant P.A., Florens L., Workman J.L., Washburn M.P. (2011). Combinatorial depletion analysis to assemble the network architecture of the SAGA and ADA chromatin remodeling complexes. Mol. Syst. Biol..

[29] Han Y., Luo J., Ranish J., Hahn S. (2014). Architecture of the Saccharomyces cerevisiae SAGA transcription coactivator complex. EMBO J..

[30] Setiaputra D., Ross J.D., Lu S., Cheng D.T., Dong M.Q., Yip C.K. (2015). Conformational flexibility and subunit arrangement of the modular yeast Spt-Ada-Gcn5 acetyltransferase complex. J. Biol. Chem..

[31] Hsu P.C., Chao C.C., Yang C.Y., Ye Y.L., Liu F.C., Chuang Y.J., Lan C.Y. (2013). Diverse Hap43-independent functions of the *Candida albicans* CCAAT-binding complex. Eukaryot. Cell.

[32] Chakravarti A., Camp K., McNabb D.S., Pinto I. (2017). The iron-dependent regulation of the *Candida albicans* oxidative stress response by the CCAAT-binding factor. PLoS One.

[33] Hortschansky P., Eisendle M., Al-Abdallah Q., Schmidt A.D., Bergmann S., Thon M., Kniemeyer O., Abt B., Seeber B., Werner E.R., Kato M., Brakhage A.A., Haas H. (2007). Interaction of HapX with the CCAAT-binding complex--a novel mechanism of gene regulation by iron. EMBO J..

[34] Coustry F., Maity S.N., Sinha S., de Crombrugghe B. (1996). The transcriptional activity of the CCAAT-binding factor CBF is mediated by two distinct activation domains, one in the CBF-B subunit and the other in the CBF-C subunit. J. Biol. Chem..

[35] Sinha S., Maity S.N., Lu J., de Crombrugghe B. (1995). Recombinant rat CBF-C, the third subunit of CBF/NFY, allows formation of a protein-DNA complex with CBF-A and CBF-B and with yeast HAP2 and HAP3. Proc. Natl. Acad. Sci. U. S. A..

[36] Sinha S., Kim I.S., Sohn K.Y., de Crombrugghe B., Maity S.N. (1996). Three classes of mutations in the A subunit of the CCAAT-binding factor CBF delineate functional domains involved in the three-step assembly of the CBF-DNA complex. Mol. Cell. Biol..

[37] Puri S., Lai W.K.M., Rizzo J.M., Buck M.J., Edgerton M. (2014). Iron-responsive chromatin remodelling and MAPK signalling enhance adhesion in *Candida albicans*. Mol. Microbiol..

[38] Baptista T., Grunberg S., Minoungou N., Koster M.J.E., Timmers H.T.M., Hahn S., Devys D., Tora L. (2017). SAGA is a general cofactor for RNA polymerase II transcription. Mol. Cell.

[39] Lenstra T.L., Benschop J.J., Kim T., Schulze J.M., Brabers N.A., Margaritis T., van de Pasch L.A., van Heesch S.A., Brok M.O., Groot Koerkamp M.J., Ko C.W., van Leenen D., Sameith K., van Hooff S.R., Lijnzaad P. (2011). The specificity and topology of chromatin interaction pathways in yeast. Mol. Cell.

[40] Bonnet J., Wang C.Y., Baptista T., Vincent S.D., Hsiao W.C., Stierle M., Kao C.F., Tora L., Devys D. (2014). The SAGA coactivator complex acts on the whole transcribed genome and is required for RNA polymerase II transcription. Genes Dev..

[41] Keaveney M., Struhl K. (1998). Activator-mediated recruitment of the RNA polymerase II machinery is the predominant mechanism for transcriptional activation in yeast. Mol. Cell.

[42] Utley R.T., Ikeda K., Grant P.A., Cote J., Steger D.J., Eberharter A., John S., Workman J.L. (1998). Transcriptional activators direct histone acetyltransferase complexes to nucleosomes. Nature.

[43] Drysdale C.M., Jackson B.M., McVeigh R., Klebanow E.R., Bai Y., Kokubo T., Swanson M., Nakatani Y., Weil P.A., Hinnebusch A.G. (1998). The Gcn4p activation domain interacts specifically *in vitro* with RNA polymerase II holoenzyme, TFIID, and the Adap-Gcn5p coactivator complex. Mol. Cell. Biol..

[44] Brown C.E., Howe L., Sousa K., Alley S.C., Carrozza M.J., Tan S., Workman J.L. (2001). Recruitment of HAT complexes by direct activator interactions with the ATM-related Tra1 subunit. Science.

[45] Bhaumik S.R., Raha T., Aiello D.P., Green M.R. (2004). *In vivo* target of a transcriptional activator revealed by fluorescence resonance energy transfer. Genes Dev..

[46] Knutson B.A., Hahn S. (2011). Domains of Tra1 important for activator recruitment and transcription coactivator functions of SAGA and NuA4 complexes. Mol. Cell. Biol..

[47] Sellam A., Askew C., Epp E., Lavoie H., Whiteway M., Nantel A. (2009). Genome-wide mapping of the coactivator Ada2p yields insight into the functional roles of SAGA/ADA complex in *Candida albicans*. Mol. Biol. Cell.

[48] Chang P., Fan X., Chen J. (2015). Function and subcellular localization of Gcn5, a histone acetyltransferase in *Candida albicans*. Fungal Genet. Biol..

[49] Tan X., Fuchs B.B., Wang Y., Chen W., Yuen G.J., Chen R.B., Jayamani E., Anastassopoulou C., Pukkila-Worley R., Coleman J.J., Mylonakis E. (2014). The role of *Candida albicans* SPT20 in filamentation, biofilm formation and pathogenesis. PLoS One.

[50] Laprade L., Boyartchuk V.L., Dietrich W.F., Winston F. (2002). Spt3 plays opposite roles in filamentous growth in *Saccharomyces cerevisiae* and *Candida albicans* and is required for *C. albicans* virulence. Genetics.

[51] Sharma S., Alfatah M., Bari V.K., Rawal Y., Paul S., Ganesan K. (2014). Sphingolipid biosynthetic pathway genes *FEN1* and *SUR4* modulate amphotericin B resistance. Antimicrob. Agents Chemother..

[52] Uhl M.A., Johnson A.D. (2001). Development of *Streptococcus thermophilus lacZ* as a reporter gene for *Candida albicans*. Microbiology.

[53] Murad A.M., Lee P.R., Broadbent I.D., Barelle C.J., Brown A.J. (2000). CIp10, an efficient and convenient integrating vector for *Candida albicans*. Yeast.

[54] Noble S.M., Johnson A.D. (2005). Strains and strategies for large-scale gene deletion studies of the diploid human fungal pathogen *Candida albicans*. Eukaryot. Cell.

[55] Moehle C.M., Hinnebusch A.G. (1991). Association of RAP1 binding sites with stringent control of ribosomal protein gene transcription in *Saccharomyces cerevisiae*. Mol. Cell. Biol..

[56] Natarajan K., Jackson B.M., Rhee E., Hinnebusch A.G. (1998). yTAF_II_61 has a general role in RNA polymerase II transcription and is required by Gcn4p to recruit the SAGA coactivator complex. Mol. Cell.

[57] Wu P.Y., Ruhlmann C., Winston F., Schultz P. (2004). Molecular architecture of the S. cerevisiae SAGA complex. Mol. Cell.

[58] Livak K.J., Schmittgen T.D. (2001). Analysis of relative gene expression data using real-time quantitative PCR and the 2^-ΔΔCT^ method. Methods.

[59] Reuss O., Vik A., Kolter R., Morschhauser J. (2004). The SAT1 flipper, an optimized tool for gene disruption in *Candida albicans*. Gene.

[60] Gola S., Martin R., Walther A., Dunkler A., Wendland J. (2003). New modules for PCR-based gene targeting in *Candida albicans*: Rapid and efficient gene targeting using 100 bp of flanking homology region. Yeast.

[61] Gillum A.M., Tsay E.Y., Kirsch D.R. (1984). Isolation of the *Candida albicans* gene for orotidine-5’-phosphate decarboxylase by complementation of *S. cerevisiae ura3* and *E. coli**pyrF* mutations. Mol. Gen. Genet..

