# Supplementary material for: Interplay between transcriptional regulators and the SAGA chromatin modifying complex fine-tune iron homeostasis
Source: J Biol Chem. 2021 Apr 29;297(1):100727. doi: 10.1016/j.jbc.2021.100727 (PMC8217685; doi:10.1016/j.jbc.2021.100727)
Supplement: Supporting information [file mmc1.pdf]

Supplementary Information for

Interplay between transcriptional regulators and the SAGA chromatin modifying complex fine-tune iron homeostasis

Manjit Kumar Srivastav*, Neha Agarwal*, Poonam Poonia, and Krishnamurthy Natarajan[†]

Laboratory of Eukaryotic Gene Regulation,

School of Life Sciences,

Jawaharlal Nehru University,

New Delhi 110067, India

*Corresponding author: Krishnamurthy Natarajan

Email: nat0200@mail.jnu.ac.in

This PDF file includes:

Supplementary text- Materials and Methods

Tables S1 to S4

References for SI

Figs. S1 to S2

Materials and Methods

Growth Conditions. *C. albicans* strains were cultured in YPD or in synthetic complete limited iron medium (SC-LIM) as described previously (1). Iron-depleted media used was either SC-LIM or YPD with 100µM BPS or 200µM BPS respectively. For iron-replete media, 100µM FAS (SC-LIM) or 200µM FAS (YPD) was supplemented to deplete media. *C. albicans* cells were cultured in either SC-LIM or YPD media and grown for 14-16h at 30°C and diluted to an initial OD of 0.25 (WT) or 0.5 (mutants) and grown for 5h and harvested.

Construction of Strains and Plasmids. The plasmids and *C. albicans* strains used in this study are provided in SI, supplemental tables S1 and S2. All strains in this study are derived from *C. albicans* parental strain SN152 and SN95(2). Oligonucleotides used in this study are listed under supplemental table S3.

Details regarding construction of plasmids and *C. albicans* strains are available on request.Briefly, genes encoding the HAP complex subunits Hap2, Hap3 and Sef1 were genomically TAP-tagged in *C. albicans* strain SN152 using the plasmids Ip21 and Ip22 (3) as DNA templates and ONC645/646, ONC649/650, ONC637/638 respectively as primers and the tagging cassettes were amplified by PCR using Phusion HF DNA polymerase. Correct integration of the tagging cassettes in *C. albicans* strains KNC398, KNC410 and KNC379 was confirmed by PCR and protein expression was confirmed by western blotting using rabbit polyclonal anti-TAP antibody (Thermo Fisher). The Hap5-TAP strain RPY453 was described previously (1). The mutant strains KNC430, KNC453 and RPY471 were constructed by deleting *sef1*Δ, *hap2*Δand *hap43*Δ in strain RPY453. The mutant strain KNC402 was constructed by TAP tagging the *SEF1* gene in strain RPY431. The various deletions were confirmed by PCR and the expression of TAP-tagged proteins by western blotting.

The strains KNC692 (WT) and KNC693 (*hap2*Δ) bearing Hap5-TAP and Hap3-FLAG was constructed by integration of the His_6-$FLAG_3$ cassette from plasmid pSH26-5 (1) into the

HAP3 locus in strains RPY453 and KNC453 respectively. The strain KNC553 bearing Hap2-TAP and Hap5-FLAG was constructed by integration of the His_6-$FLAG_3$ cassette from plasmid pSH26-5 (1) into the *HAP5* locus in strain KNC398. The strain KNC558 was constructed in multiple steps as follows. First, the *HAP5* locus was FLAG-tagged in strain RPY431, and subsequently, the *HAP2* locus was TAP-tagged to obtain strain KNC558.

To construct *gcn5*Δ/Δ, *spt7*Δ/Δ and *spt20*Δ/Δdeletion strains, we carried out sequential deletion of both alleles using the *HIS1-ARG4-HIS1* (HAH) deletion cassette from plasmid pHAH1 (4), using primers specific for *GCN5* (ONC601/ONC115 and ONC601/ONC114), *SPT7* (ONC602/ONC115 and ONC603/ONC114) or *SPT20* (ONC605-ONC115 and ONC606-ONC114) and amplified the respective up-split and down-split fragments, combined and transformed into *C. albicans* strain SN95 and selected for Arg^+ colonies. The correct integrations were confirmed using PCR, and the resultant heterozygous strains KNC641, KNC651 and KNC653 were grown non-selectively in YPD and Arg^+ His^+ colonies representing homozygous deletion strains KNC642, KNC652 and KNC654 were selected, and confirmed by PCR.

For reintegration of *GCN5*, *SPT7* and *SPT20*into the homozygous deletion strains KNC642, KNC652 and KNC654, plasmid pNIM1 was sequentially digested with BglII/MluI to obtain nourseothricin resistance marker CaSAT1, while plasmid pCIp10 was digested with NotI/XbaI and end filled with Klenow. Then linearized plasmid pCIp10 and insert CaSAT1 was ligated and transformed to elctrocompetent bacterial cells DH10B to obtain plasmid pCIp10-SAT1 (pPI1). The coding sequences along with 1000bp upstream and 500bp downstream of ORF sequences were amplified using primer pairs ONC1156-ONC1157 (*GCN5*), ONC1154-ONC1155 (*SPT7*) and ONC1152-ONC1153 (*SPT20*). The amplified products were digested with BamHI (for *SPT7* and *SPT20*) or SacI (for *GCN5*) and cloned into plasmid pPI1 to obtain plasmid pPI2 (*GCN5*), pPI3 (*SPT7*) and pPI4 (*SPT20*).

The plasmids pPI2, pPI3 and pPI4 were linearized with PmeI, BstEII and AflII respectively and transformed into KNC642, KNC652 and KNC654strains and positive transformants were confirmed by PCR using ORF-specific primer pairs ONC661/ONC662, ONC663/ONC664, ONC708/ONC709 and obtainedPRI1 (*gcn5Δ/GCN5*), PRI2 (*spt7Δ/SPT7*) and PRI3 (*spt20Δ/SPT20*) respectively.

Strains for β-Gal assays were constructed as follows. The P_{ACT1}*::lacZ-ACT1t*insert from plasmid pAU36 (5) was subcloned into CIp10 (6) to construct pNA36. A 1kb *HAP43* promoter region was amplified from SC5314 strain genomic DNA and cloned as a as a KpnI-SalI fragment into the KpnI-XhoI digested pNA36 to replace P_{ACT1} and obtained plasmid pNA40. The plasmid pNA40 was linearized with StuI and transformed into SN152, RPC75 andTF015 (*sef1Δ*) by electroporation to integrate the reporter plasmid at the *RPS1* locus and obtained KNC332, KNC340 and KNC348. The correct integration was confirmed by PCR.

The strains bearing wild-type and mutant *FRP1* promoters were constructed in several steps. First, the wild-type *FRP1* coding sequence along with 500bp upstream region was deleted in strains RPY453 and KNC379 to obtain strains KNC467 and KNC481 respectively. The homozygous *frp1Δ*deletion was confirmed by PCR. Next, a DNA fragment containing the *FRP1* coding sequence flanked by 1kb upstream region and 300bp downstream region was amplified as a KpnI-SacI fragment using ONC630-ONC631 and cloned into pLitmus28 to obtain pNA51. The wild-type *FRP1* fragment was then subcloned into pSN69 (2) and obtained plasmid pNA61, which was integrated at the native *FRP1* locus in strain KNC467 and KNC481 and obtained strains KNC479 and KNC486 respectively. The correct integration was confirmed by PCR.

To construct point mutations in the HAP complex binding site (CCAAT motif) in the *FRP1* promoter, the mutagenic oligonucleotide pair ONC901-ONC902 was designed using the Quick Change Primer Design tool (Agilent Technologies) and the CCAAT motif (-134 with

reference to ATG) was mutated to TGCGC as per Baek et al. (7). The plasmid pNA61 was used as a template along with the mutagenic primers and amplified using the Quick Change II XL Site Directed Mutagenesis Kit and obtained plasmid pNA68. Similarly, the putative Sef1 binding site (8) was mutated using the mutagenic oligonucleotides ONC929-ONC930 and ONC931-ONC932 and obtained plasmid pNA76. The wild-type and the two mutant insert sequences in the plasmid clones were verified by Sanger sequencing. Next, the plasmids pNA68 and pNA76 were linearized with Bpu101, gel-purified and integrated at the chromosomal *frp1Δ/Δ* locus in strains KNC467 and KNC481 and obtained strains KNC494, KNC498, KNC505 and KNC509. The correct integrations were confirmed by PCR.

β-galactosidase assay. Briefly, three independent transformants of each strain were grown overnight in SC-LIM and then diluted to 30ml SC-LIM + 100 μM BPS (-Fe) at a starting density of 0.3 OD600 and 0.5 OD_{600} for WT and the mutant strains (*hap43Δ/Δ* and *sef1Δ/Δ*), respectively. For iron replete conditions (+Fe; SC-LIM + 100 μM BPS and 100 μM FAS), cells were diluted to a starting density of 0.25 OD_{600} for all the strains and grown for 5 hrs till ~1.0 OD. Cells were harvested and cell pellets washed with Z-buffer (9), re-suspended in 200µl Z-buffer with 2mM PMSF and vortexed for 2 min in cold room using a Braun homogenizer. Supernatants were collected and protein extracts were cleared by centrifugation at 13,000 rpm at 4°C for 15 min. The total protein concentration in the cell lysates was determined by Bradford assay using Softmax Pro 2.6 program in SpectraMax 340 microplate reader. The β-galactosidase kinetic assay was performed at 37°C in 96-well microplates in SpectraMax 340 microplate reader with ONPG as substrate (0.75 mg/ml), absorbance monitored, and the β-galactosidase specific activity was calculated as described previously (10) and plotted using GraphPad Prism 6. Statistical analysis of Hap43 protein level and the β-galactosidase activity was performed by comparing expression in -Fe compared to +Fe and and statistical significance determined by Student's T-test.

Whole Cell Extract Preparation and Western blotting. The cell pellets were resuspended in chilled Winston Buffer (40mM HEPES-NaOH pH 7.5, 350mM NaCl, 10 % glycerol, 0.1% Tween 20 (11) with added protease inhibitors (2.5µg/ml Aprotinin, 2mM Benzamidine, 1mM Dithiothreitol, 2µg/ml Leupeptin, 2µg/ml Pepstatin, 100µM PMSF, 10µg/ml TPCK, 10µg/ml TLCK), and vortexed in presence of glass beads. The lysates were cleared by centrifugation at 13,000 rpm for 15 min at 4°C. Cell lysates were resolved on 10% SDS-PAGE, transferred to nitrocellulose membrane, and probed with different antibodies, and detected with ECL-Prime Western blot detection reagent (GE Healthcare) and exposed to X-Ray film.

RNA analysis. TotalRNAwasisolated from *C. albicans* cells and cDNA was synthesized essentially as described before (1). Real-time qRT-PCR was carried out in Applied Biosystems Fast 7500 Real-time PCR system using gene-specific primers and differential expression was calculated by the comparative $C_T$method (12). The *SCR1* RNA, an RNA polymerase III transcript was used as endogenous control (1).

Chromatin Immunoprecipitation (ChIP) Assay. ChIP assayswere conducted essentially as described before (1). Briefly, all strains were pre-cultured overnight in SC-LIM medium, grown in fresh SC-LIM medium with 100µM BPS alone or with BPS and 100µM FAS for 5 hours at 30 °C to an OD_{600} of 0.8–1.0, cross-linked with 1% (v/v) formaldehyde,quenched with 125mM glycine, cells harvestedand chromatin extracts prepared by shearing in Bioruptor (model UCD 300, Diagenode). For chromatin immunoprecipitation of TAP-tagged proteins, the sheared chromatin extractsequivalent to ~25OD_{600} cells from control and experimental strains were immunoprecipitated with about 25 µl of IgG-Sepharose 6 Fast Flow (pre-blocked with 0.2mg/ml sheared Calf thymus DNA and 0.1% (w/v) BSA) for 3h at 4°C. For Hap43 ChIP, 2µl affinity-purified antibody (1) was bound to 30µl beads and immunoprecipitated for 4h at 4°C. The RNA pol II, histone H3 and H3-K9Ac chromatin immunoprecipitations were conducted using chromatin extract from ~35 OD600 equivalent cells and 2µgeach of total H3, H3-K9Ac and RNA polymerase II antibody pre-bound toDynabeads Protein G.

Immunoprecipitation was carried out for 9h at 4°C, washed and eluted in 25µl 0.1xTE. The input DNA and immunoprecipitated DNA were purified, and diluted 1:10,000 (input DNA) and 1:5 (IP DNA), and probed by quantitative real-time PCR for specific regions of interest as well as for the control non-specific region (ca21chr1_1573500–1574000). For all chromatin immunoprecipitations, enrichment was calculated for target regions and for the control non-specific region (ca21chr1_1573500–1574000) and specific enrichment calculated with respect to input total chromatin. For the TAP chromatin immunoprecpitations, the specific enrichment was determined by further normalizing the background amount of immunoprecipitation obtained in mock immunoprecipitation conducted using chromatin extracts from untagged strain. Similarly, for Hap43 chromatin immunoprecpitations, the specific enrichment was determined by further normalizing the background amount of immunoprecipitation obtained in the *hap43Δ* mutant chromatin immunoprecipitation.

Table S1. List of plasmids used in this study

Plasmid	Description	Source
pLITMUS28	Cloning vector, Amp^R	NEB
pSN69	*C.d. ARG4*	(2)
pHAH1	*HIS1-ARG4-HIS1*	(4)
pAU36	P_{ACT1}*::lacZ ACT1T*	(5)
pSH26-5	*C.a.SAT1* HIS_6-$FLAG_3$*-ACT1t*	(1)
pSFS2A	*SAT1* Flipper Cassette	(13)
pFA-HIS1	*C.d. HIS1*	(14)
pFA-LEU2	*C.m. LEU2*	(14)
CIp10- *CaURA3*	pCIp10 with *C.a.URA3*	(6)
pNIM1	*C. a. SAT1CaADH1*pTet-*CaGFP*	
CIp10-*CdARG4*	pCIp10 with *C.d. ARG4* in place of *C.a.URA3*	(1)
pRC20	*HAP43* in CIp10 *C.d. ARG4*	(1)
Ip21	TAP tag, *C.m. LEU2*	(3)
Ip22	TAP tag, *C.d. HIS1*	(3)
pNA36	P_{ACT1}*-LacZACT1t*inCIp10-*CdARG4*	This work
pNA40	$P_{HAP43\ (1\ Kb)}$ *lacZ-ACT1t*in CIp10-*CdARG4*	This work
pNA51	$P_{FRP1(1kb)}$ *FRP1* $_{3'UTR(300bp)}$ in pLITMUS28	This work
pNA60	$P_{FRP1(1kb)}$ *FRP1* $_{3'UTR(300bp)}$ in CIp10	This work
pNA61	$P_{FRP1(1kb)}$ *FRP1* $_{3'UTR(300bp)}$ into pSN69	This work
pNA68	$P_{FRP1(1kb)\ CCAATmut}$*FRP1* $_{3'UTR(300bp)}$ in pSN69	This work
pNA72	$P_{FRP1(1kb)\ CGG(I)mut}$*FRP1* $_{3'UTR(300bp)}$ in pSN69	This work
pNA76	$P_{FRP1(1kb)\ CGG(I)mut\ CGG(II)mut}$*FRP1* $_{3'UTR(300bp)}$ in pSN69	This work
pPI1	*pCIp10* with *Ca SAT1*	This work
pPI2	*GCN5* in *pCIp10-Ca SAT1*	This work
pPI3	*SPT7* in *pCIp10-Ca SAT1*	This work
pPI4	*SPT20* in *pCIp10-Ca SAT1*	This work

Table S2. List of *C. albicans* strains used in this study

Strain	Parent	Relevant Genotype	Source
SC5314	-	Wild-typeclinical isolate	(15)
SN152	RM1000	*arg4Δ/arg4Δ/leu2Δ/leu2Δ/his1Δ/his1ΔURA3/ura3Δ::*imm^{434}*IRO1/ iro1Δ::*imm^{434}	(2)
SN95	RM1000	*arg4Δ/arg4Δ/his1Δ/his1ΔURA3/ura3Δ::*imm^{434}*IRO1/ iro1Δ::*imm^{434}	(2)
TF087	SN152	*hap2Δ::C.m.LEU2/hap2Δ::C.d.HIS1*	(16)
TF015	SN152	*sef1Δ::C.m.LEU2/sef1 Δ::C.d.HIS1*	(16)
TF093	SN152	*hap5Δ::C.m.LEU2/hap5Δ::C.d.HIS1*	(16)
RPC75	SN152	*hap43Δ::C.m.LEU2/hap43Δ::C.d.HIS1*	(1)
RPY 431	SN152	*hap3Δ::FRT/hap3Δ::FRT*	(1)
RPY 453	SN152	*HAP5::TAP-CdHIS1/HAP5::TAP-CmLEU2*	(1)

RPY 552	SN152	*HAP5/HAP5::FLAG*$_3$*-HIS*$_6$*-SAT1-FLP*	(1)
RPY471	RPY453	*hap43Δ:SAT1-FLP/hap43Δ::FRT HAP5::TAP-CdHIS1/HAP5::TAP-CmLEU2*	This work
KNC332	SN152	<pNA40>	This work
KNC340	RPC75	*hap43Δ/ hap43Δ*<pNA40>	This work
KNC348	TF015	*sef1Δ/ sef1Δ*<pNA40>	This work
KNC369	SN152	*SEF1::TAP-CdHIS1/SEF1*	This work
KNC379	SN152	*SEF1::TAP-CdHIS1/SEF1::TAP-CmLEU2*	This work
KNC398	SN152	*HAP2::TAP-CdHIS1/HAP2::TAP-CmLEU2*	This work
KNC402	RPY431	*hap3Δ::FRT/hap3Δ::FRT SEF1/SEF1::TAP-CmLEU2*	This work
KNC410	SN152	*HAP3::TAP-dHIS1/HAP3::TAP-CmLEU2*	This work
KNC430	RPY453	*HAP5::TAP-CdHIS1/HAP5::TAP-CmLEU2 sef1::FRT/sef1::FRT*	This work
KNC453	RPY453	*HAP5::TAP-CdHIS1/HAP5::TAP-CmLEU2 hap2Δ::FRT/hap2Δ::FRT*	This work
KNC454	RPY453	*hap2Δ::FRT/hap2Δ::FRT HAP5-TAP-CmLEU2/HAP5-TAP-CdHIS1*	This work
KNC467	RPY453	*HAP5::TAP-CdHIS1/HAP5::TAP-CmLEU2 frp1Δ::FRT/frp1Δ::FRT*	This work
KNC479	KNC467	*HAP5::TAP-CdHIS1/HAP5::TAP-CmLEU2 frp1Δ::FRT/frp1Δ::FRT pSN69-ARG4-FRP1*	This work
KNC481	KNC379	*SEF1::TAP-CdHIS1/SEF1::TAP-CmLEU2 frp1Δ::FRT/frp1Δ::SAT1-FLP*	This work
KNC486	KNC481	*SEF1::TAP-CdHIS1/SEF1::TAP-CmLEU2 frp1Δ::FRT/frp1Δ::SAT1-FLP pSN69-ARG4-FRP1*	This work
KNC494	KNC467	*HAP5::TAP-CdHIS1/HAP5::TAP-CmLEU2 frp1Δ::FRT/frp1Δ::FRT pSN69-ARG4-FRP1*$_{TGCGC}$	This work
KNC498	KNC481	*SEF1::TAP-CdHIS1/SEF1::TAP-CmLEU2 frp1Δ::FRT/frp1Δ::SAT1-FLP pSN69-ARG4-FRP1*$_{TGCGC}$	This work
KNC505	KNC467	*HAP5::TAP-CdHIS1/HAP5::TAP-CmLEU2 frp1Δ::FRT/frp1Δ::FRT pSN69-ARG4-FRP1*$_{CGAGGTATC}$	This work
KNC509	KNC481	*SEF1::TAP-CdHIS1/SEF1::TAP-CmLEU2 frp1Δ::FRT/frp1Δ::SAT1-FLP pSN69 -ARG4- FRP1*$_{CGAGGTATC}$	This work
KNC553	KNC398	*HAP2::TAP-CdHIS1/HAP2::TAP-CmLEU2 HAP5-FLAG-SAT1-FLP/HAP5*	This work
KNC555	RPY431	*hap3Δ::FRT/hap3Δ::FRT HAP5-FLAG-SAT1-FLP/HAP5*	This work
KNC558	KNC555	*hap3Δ::FRT/hap3Δ::FRT HAP5-FLAG-SAT1-FLP/HAP5 HAP2-TAP-CmLEU2/HAP2*	This work
KNC560	RPY431	*hap3Δ::FRT/hap3Δ::FRT HAP2-TAP-CmLEU2/HAP2*	This work
KNC641	SN95	*gcn5Δ::HAH1/GCN5*	This work
KNC651	SN95	*spt7Δ::HAH1/SPT7*	This work
KNC653	SN95	*spt20Δ::HAH1/SPT20*	This work
KNC642	KNC641	*gcn5Δ::HAH1/gcn5Δ::HIS1*	This work
KNC652	KNC651	*spt7Δ::HAH1/spt7Δ::HIS1*	This work

KNC654	KNC653	*spt20Δ::HAH1/spt20Δ::HIS1*	This work
KNC691	SN152	*HAP3-FLAG-SAT1-FLP /HAP3*	This work
KNC692	RPY453	*HAP3-FLAG-SAT1-FLP/HAP3 HAP5-TAP-CmLEU2/HAP5-TAP- CdHIS1*	This work
KNC693	KNC454	*hap2Δ::FRT/hap2Δ::FRT HAP3-FLAG-SAT1-FLP/HAP3 HAP5-TAP-CmLEU2/HAP5-TAP- CdHIS1*	This work
PRI1	KNC642	*gcn5Δ::HAH1/gcn5Δ::HIS1<pPI2>*	This work
PRI2	KNC652	*spt7Δ::HAH1/spt7Δ::HIS1<pPI3>*	This work
PRI3	KNC654	*spt20Δ::HAH1/spt20Δ::HIS1<pPI4>*	This work

Table S3. List of oligonucleotides used in this study

Primer	Sequence	Notes
ONC30	5'-ATTTCTTCCATGGTTAACAG-3'	Universal primer in the *HIS1* containing cassette
ONC33	5'-GATATCATTTCCGATGAAGC-3'	Universal primer in the *LEU2* containing cassette
ONC45	5'-TTTGAAGTCGACATGCCCGCAAAAG-3'	-12 to +13 w.r.t. ATG. Fwd primer for amplifying *HAP43* gene
ONC56	5'-AAT TAAAGGTGGAGAACC-3'	+404 to +421 w.r.t. ATG of orf19.681
ONC58	5'-AACTTTTTATGACGAGAC-3'	+1790 to +1773 w.r.t. ATG of orf19.681
ONC109	5'-TGGATAACAAACCGTTCTTC-3'	Reverse Primer in *LEU2* gene
ONC110	5'-CGGTGGCACATTTCACAC-3	Reverse Primer in *HIS1*gene
ONC114	GGTGCCACTGATCCATTG	Position 61 to 78 within Ca*ARG4ORF* (Ca*ARG4*-F61)
ONC115	GCCAACATATCCATAGTTAAAGC	Position 1108 to 1130(c) within *CaARG4ORF* (Ca*ARG4*-R1130)
ONC140	5'-TCTTGGTGAGAACAGCGACCGAAA-3'	Reverse primer for upstream split marker of *SAT1* flipper
ONC141	5'-GGAGCGATAAGCGTGCTTCTGCCG-3'	Fwd primer for downstream split marker of *SAT1* flipper
ONC145	5'-CGTATTCACTTAATCCCACAC-3'	Rev Primer binding to 3'UTR of *RPS1*
ONC243	5'TTTCAACAAAGTCAACCACCGTTTATGAATTTAACGCTATAACAAT CATTTAATATATTAGAAGCTTCGTACG CTGCAGGTC-3'	Fwd Primer for disrupting *SFU1*
ONC244	5'ACTATACACGAAAAAACTTCATAATACCTATATATATATATACAAC CCTTGATATTAACTTCTGATATCATC GATGAATTCGAG-3'	Rev Primer for disrupting *SFU1*
ONC247	5'-ATTTGCAGCTGGTTCAAC-3'	Rev Downcheck Primer for *SFU1*
ONC248	5'-CGGTACCGACGTGATCACCTGGTA-3'	Rev diagnostic Primer in *C.m.LEU2*
ONC249	5'-TCTAAACTGTATATCGGCACCG-3'	Rev diagnostic Primer in *C.d.HIS1*
ONC257	5'AGATGAAAACAAGCCAATCAAGCAAGAAGAACATCGGAGTGCACT TTCTATTGGGAAGTTGTTAAATGGAGGATCCATGGAAAAGAGA-3'	Fwd Primer for Sfu1-TAP tagging
ONC258	5'-TTCCGATTTTCAATTTTGTTACACAAACATAAATC ATGCTCATTAAATGCAAGAAAGGGGATTGTTTTGCGTGTGATGGATA TCTGCA-3'	Rev Primer for Sfu1-TAP tagging
ONC263	5'-AAACAGCTATGACCATGA-3'	Sequencing Primer in CIp10-*ARG4* vector
ONC267	5'-GGCGGAAAATGCAAATGGTCATAATGATGAAGCT ACGTATGAAAATTTTAACGGTTACCAAAACAATTATGGATCCATGG AAAAGAGA-3'	Fwd Primer for Hap5-TAP tagging
ONC268	5'-CTAATATCCGAGTGCTTCCGATGAAACAAACCCA CCGAAATATCAACTATGTAATCAAGTGAGGAACTGAGTGTGATGG ATATCTGCA-3'	Rev Primer for Hap5-TAP tagging

ONC269	5'-TACTTGCTCTTGTGGCTCAAAG-3'	Diagnostic Rev. primer binding to 3'-UTR of *Ca HAP5*
ONC 470	5’-TTCAGTAGGTACCGCTGACACAAACGGGAACAGA AATA-3’	FwdKpnI site appended to amplify *HAP43* (1kb) promoter region
ONC471	5’-GGCAGCTGTCGACGTTGTTCAAATTGAAATTCTAA TTA-3’	Rev SalI site appended to amplify *HAP43* (1kb) promoter region
ONC574	5’-TTCATTAGGTACCCCAACTAGCCACGTCGTTCCC-3’	Rev SalI site appended to amplify *HAP43*(-282 bp) promoter region
ONC575	5’-TTCATTAGGTACCCATCAGCAAATGAAGTTGTGT-3’	Rev SalI site appended to amplify *HAP43* (-369 bp) promoter region
ONC 595	5'-CAGTCATGTTCATGGAC -3'	Rev sequencing primer in the *LacZ* gene in pAU36
ONC600	5’-CGGAAAGCTGAAGAACATTTAAACCCCAAACAAGCAATAACCG ATCAATAACAATTCGTACTTCGTACGCTGCAGGTC-3’	Forward long primer for knock out construction of *GCN5* by HAH cassette
ONC601	5’-AATTTCAGTACT ATATGGTATTTCGGCCAAGAATCACATATCATATATTCGTCAAAAAG CTGATATCATCGATGAATTCGAG-3’	Reverse long primer for knock out construction of *GCN5* by HAH cassette
ONC602	5’-CTTATACATATTATTTACACCAACAATAAATTTTCCTTTTTCTTA ATATTGGATTGAAATCTTCGTACGCTGCAGGT-3’	Forward long primer for knock out construction of *SPT7* by HAH cassette
ONC603	5’-ATTCGAGTGGGGGTAGTTGGTTGGTTGTGTAAGATATCAATATC AGGTAAAGTATTATTTCTGATATCATCGATGAATTCGAG-3’	Reverse long primer for knock out construction of *SPT7* by HAH cassette
ONC604	5’-CAATCATAAACAAATAGTCATATTAAATAGATTCATTAAGACTA CCTTTGACATTACACTCTTCGTACGCTGCAGGTC	Forward long primer for knock out construction of *SPT20* by HAH cassette
ONC605	TTTCATAGTGTTTGCTGAATCATAAGCATAAAATACAAAGGCAAACT TCTATAAGTTACACTGATATCATCGATGAATTCGAG-3’	Reverse long primer for knock out construction for *SPT20* by HAH cassette
ONC 630	5’-CGTCCAGGTACCAAGAAGGTACATCATGAA-3’	KpnI site appended Fwd primer to amplify P_{FRP1} (1Kb) *FRP1*$_{3'UTR(300bp)}$
ONC 631	5’-AATACTGAGCTCCAACACAATGCCACACTT-3’	SacI site appended Rev primer to amplify P_{FRP1} (1Kb) *FRP1*$_{3'UTR(300bp)}$
ONC 632	5'-CCAGCCCATTGTGGCATCACGCAGAAACATCCTGT ATCCCAATATGGACAAGGGGTGTTACTTGTACTGAGAAGTTCCTATA CTTTCTAG-3'	Fwd primer for deleting-500 bp and *FRP1* using *SAT1-FLP*
ONC 633	5’-GTCCTTACGTACAGGAATTTATGTCCGCCTTGCTA AAACATACACAAACCTTAAATTTATCCTATATACCGAAGTTCCTATT CTCTAGAA-3’	Rev primer for deleting-500 bp and *FRP1* using *SAT1-FLP*
ONC 637	5’-ATGGTTTAATAATGATAACCAAGATGACGACTTTT TGGGTTGGTTTGATGTTAATATGATGCAAGAGAAAGGATCCATGGA AAAGAGA-3’	Fwd primer *SEF1* TAP tagging
ONC 638	5'-TAACTTAAAGTTTTAATTAATTTATTTATACTAACG TTAACTTGGTAACCCTTAGTGAGGCCAGCTAATTGTGTGATGGATAT CTGCA-3'	Rev primer *SEF1* TAP tagging
ONC 639	5'-ACCGCAATTTTCAATGAT -3'	FwdUpChk diagnostic primer for *SEF1* TAP tagging
ONC640	5'-TAGCACTTATCCATCTGAAC-3'	Rev DownChk diagnostic primer for *SEF1* TAP tagging
ONC645	5’ATCAATAAAGAATGATAATAACAATAACAATAATAATAATGGTAA TGATCTACAAGAAGAAGAAGAACGAGGATCCATGGAAAAGAGA-3’	Fwd Primer *HAP2* TAP tagging
ONC646	5’-AAATTGAAAATTGAAAAAAAAATTATGAAAGATT CATTAGGATAAAAATTAACATTAACACTTTTTGAACGTGTGATGGAT ATCTGCA -3’	Rev Primer *HAP2* TAP tagging
ONC647	5’-GAGAATGGGAATGAAATCAAA-3’	FwdUpchk diagnostic primer for *HAP2* TAP tagging
ONC648	5'-AAAGAATCAATCGATCAATCA-3'	Rev Downchk diagnostic primer for *HAP2* TAP tagging
ONC649	5’-CAAAACGACAAACAACGGATTATTCCTGATTTACT TAACATAACTAATTCATATTCTGAAGATGAATTTATTGGATCCATGG AAAAGAGA-3’	Fwd Primer *HAP3* TAP tagging
ONC650	5’-AAAACCTAAATCTCTAAACTATAGATGTAAACAA AATCTTAAATTCACAATTTTATTACAATATAAATTAGTGTGATGGAT ATCTGCA-3’	Rev Primer *HAP3* TAP tagging
ONC651	5’-GATGATGAAGATGAAGAAGAA-3’	Fwd Upchk diagnostic primer for *HAP3* TAP tagging
ONC652	5'-TAATTGAGATTTCAGTGAGG-3'	Rev Downchk diagnostic primer for *HAP3* TAP tagging
ONC661	GGTGATAAGTCAGGAGCCACGG	Forward ORF specific primer for *GCN5*
ONC662	CGGTAACATCGAGCACTGCATC	Reverse ORF specific primer for *GCN5*
ONC663	CAACGAGAGTGCTGGTGGTGA	Forward ORF specific primer for *SPT7*

ONC664	ATTCGGTGTCTCTGCTTCTGCT	Reverse ORF specific primer for *SPT7*
ONC708	TCAACATCGTCTCCATCCAAA	Forward ORF specific primer for *SPT20*
ONC709	TGAAGAGTCTGTTGGGGATTT	Reverse ORF specific primer for *SPT20*
ONC 752	5'-ATCATAATTATATTTGAATTATTTGATTCTACTAC CAAAAGTTAAACAACCAAGAAATCAACAAACCGAAGAAGTTCCTAT ACTTTCTAG-3'	Fwd primer for *HAP2* deletion using *SAT1-FLP* cassette
ONC 753	5'-TTCTAAATCGTGTTCTCACTTGTCTTGAAGAATGA AATAGAATTCTTGTTAATGTTGTTATTGTTATTGAGAAGTTCCTATTC TCTAGAA-3'	Rev primer for *HAP2* deletion using *SAT1-FLP* cassette
ONC 754	5'-GAATTCAGTAAACAAACATTCAATTAAGACGAAG AAATACAGAAAGACCCCCCAAAACAATACTCAAACAGAAGTTCCTA TACTTTCTAG-3'	Fwd primer for *HAP5* deletion using *SAT1-FLP* cassette
ONC 755	5'-TCTAGGAATTAACTATTATTACAAAATCAAACACT ATTTTTAAAATGAACGAAAAAAAAAAAAAAAATCCTGAAGTTCCTAT TCTCTAGAA-3'	Rev primer for *HAP5* deletion using *SAT1-FLP* cassette
ONC 756	5'-TGTGTCGACAACCAATCGACTCCTCCCTTAACTAG AACCATAGAACCTCAACATTTGTTTCTATAGAAAAGAAGTTCCTATA CTTTCTAG-3'	Fwd primer for *SEF1* deletion using *SAT1-FLP* cassette
ONC 757	5'-ACTAAACTTATTCATTACAAAATCATATTAACATA ATTACTAACTATTTACATTCTAATGAGGTAGAATCGAAGTTCCTATT CTCTAGAA-3'	Rev primer for *SEF1* deletion using *SAT1-FLP* cassette
ONC 758	5'-CAACGGACGATGCAGAAAGTAGTT-3'	FwdUpchk diagnostic primer *SEF1* deletion
ONC 759	5'-ATGTGGTGTCAATAGCACTTATCC-3'	Rev Downchk diagnostic primer *SEF1* deletion
ONC 760	5'-GGTCACAAATCCAATCATTG-3'	Fwd primer to check *SEF1* trisomy
ONC 761	5'-ACCGTCTGGTCTAGTAAC-3'	Rev primer to check *SEF1* trisomy
ONC 762	5'-GCTTCAACCGATGATCGCA-3'	Fwd primer to check *HAP3* deletion
ONC 763	5'-GATCAACAGATAGAGAAGGGAG-3'	Rev primer to check *HAP3* deletion
ONC 764	5'-CATGTGAGGAGAAGTATCG-3'	Fwd primer to check *HAP3* trisomy
ONC 765	5'-GTTTTGGCGTTGATCATT-3'	Rev primer to check *HAP3* trisomy
ONC 766	5'-GAAACTCCGATATCGCCAAACAAA-3'	Fwd primer to check *HAP2* deletion
ONC 767	5'-CAATGTGTGGATGTGATTCAAATT GAT-3'	Rev primer to check *HAP2* deletion
ONC 768	5'-TCATCATCATGTTGGTTC-3'	Fwd primer to check *HAP2* trisomy
ONC 769	5'-TGATGACTGTTCATTCTCA-3'	Rev primer to check *HAP2* trisomy
ONC 770	5'-TACTGAGGTACCTAGTCTCCGATTTCATTCCAT-3'	Rev SalI site appended to amplify *HAP43* (-618 bp) promoter region
ONC 804	5’-ATGAAGTTTGAAAAAGGTAAAGTGAGAATTTTGC CTAAACCATCCCCTACACCAACCAACCCACAAACCCGAAGTTCCTAT ACTTTCTAG-3’	Fwd primer SII for *SEF1* deletion using *SAT1-FLP* cassette
ONC 805	5'-TTATTTCTCTTGCATCATATTAACATCAAACCAAC CCAAAAAGTCGTCATCTTGGTTATCATTATTAAACGAAGTTCCTATT CTCTAGAA -3'	Rev primer SII for *SEF1* deletion using *SAT1-FLP* cassette
ONC821	5’-ATGACATCACTAGAAGATCCAAATGGACAAGCAC AAGCAACTTATTTTCAAGAGTCAGTTTCTACAACTGGAAGTTCCTAT ACTTTCTAG-3’	Fwd primer SII for *HAP2* deletion using *SAT1-FLP* cassette
ONC822	5'-TTATCGTTCTTCTTCTTCTTGTAGATCATTACCATT ATTATTATTGTTATTGTTATTATCATTCTTTATTGAAGTTCCTATTCTC TAGAA-3'	Rev primer SII for *HAP2* deletion using *SAT1-FLP* cassette
ONC 838	5’-ATGAACGAAGATCCACAGTCCGAAATTATGGAAA GATACAATGAATCAGCTTATTTGAGAGACGACCAGCGAAGTTCCTAT ACTTTCTAG -3’	Fwd primer SII for *HAP5* deletion using *SAT1-FLP* cassette
ONC 839	5'-CTAATAATTGTTTTGGTAACCGTTAAAATTTTCAT ACGTAGCTTCATCATTATGACCATTTGCATTTTCCGAAGTTCCTATT CTCTAGAA-3'	Rev primer SII for *HAP5* deletion using *SAT1-FLP* cassette
ONC 850	5’-AGTAGCCAAGAATCTGGCACTAGCAACATCTTCA CCCTTACACTTTTGAGCAACACATTGACCAACTAAAGAAGTTCCTAT ACTTTCTAG-3’	Fwd primer for $P_{FRP1(500)}$ *FRP1* deletion using *SAT1-FLP* cassette
ONC 851	5'-CTAAAACGACTCTGTATAACAATACACTTCCGGTG CACCTTTCACCCTTCTAGCACTTTGACAATGATGTGAAGTTCCTATTC TCTAGAA -3'	Rev primer for $P_{FRP1(500)}$ *FRP1* deletion using *SAT1-FLP* cassette
ONC 852	5'-GATGTGCTGCAAGGCGATTA-3'	Fwd primer in *FRP1* promoter region
ONC 854	5'-TTATCCACAATCAAGGAAACAG-3'	Fwd primer in *FRP1* coding region
ONC 901	5'-CAGACAAGGGATGCGGCCATCGTTTAGCGCATGG ATTT ATCGTTTCTATGCCAAGAATC-3'	Sense primer to mutate CCAAT box in *FRP1* promoter
ONC 902	5'-GATTCTTGGCATAGAAACGATAAATCCATGCGCT AAAC GATGGCCGCATCCCTTGTCTG-3'	Antisense primer to mutate CCAAT box in *FRP1* promoter
ONC 929	5'-GGTGGATTTATCGTTTCTATACCAAGAATTCGAAA ACTGCGAAATTTTACGCAGCAC-3'	Sense primer to mutate 1st CGG repeat for Sef1 binding in *FRP1* promoter

ONC 930	5'-GTGCTGCGTAAAATTTCGCAGTTTTCGAATTCTTG GTAT AGAAACGATAAATCCACC-3'	Antisense primer to mutate 1st CGG repeat for Sef1 binding in *FRP1* promoter
ONC 931	5'-CGATAAATCCACCAATTAAACGATGGATCCATCCC TTGTCTGATGATAATAAGG-3'	Sense primer to mutate 2nd CGG repeat for Sef1 binding in *FRP1* promoter
ONC 932	5'-CCTTATTATCATCAGACAAGGGATGGATCCATCGT TTAATTGGTGGATTTATCG-3'	Antisense primer to mutate 2nd CGG repeat for Sef1 binding in *FRP1* promoter
ONC 1107	5-CAAAACGACAAACAACGGATTATTCCTGATTTACT TAACATAACTAATTCATATTCTGAAGATGAATTTATTCATCATCACC ATCATCATGGT-3	Fwd Primer *HAP3* FLAG tagging
ONC 1108	5'-AAAACCTAAATCTCTAAACTATAGATGTAAACAAAATCTTAAATTCA CAATTTTATTACAATATAAATTA GCTGGAGCTCCACCGCGGTG -3'	Rev Primer *HAP3* FLAG tagging
ONC1152	CTCGGATCCTGGATCGATTTGATAGACTC	Forward primer for *SPT7* wrt 1.5kb upstream of ATG
ONC1153	GAG GGA TCC TTT CTT GTG TTC TAG GCC	Reverse primer for *SPT7* wrt 500bp downstream of TAA
ONC1154	CAC GGA TCC TAA ACT GAC AAT TTA AAA CG	Forward primer for *SPT20* wrt 1kb upstream of ATG
ONC1155	CTC GGA TCC AGA TTT CAA TTA TTT AAA GG	Reverse primer for *SPT20* wrt 500bp downstream of TAA
ONC1156	CAC GAG CTC ATA ACT TGA AGA TCC CG	Forward primer for *GCN5* wrt 1kb upstream of ATG
ONC1157	GTC GAG CTC CAC TGA AGT AGA CTT CCC AC	Reverse primer for *GCN5* wrt 500bp downstream of TAA
Real time PCR Primers		
ONC176	5'-TGCTCGTGTACCTGCTGTTTG-3'	Fwd RT PCR primer for *CaSCR1*
ONC177	5'-ACACTGATTACTCTAGCGTTCAGAATTC-3'	Rev RT PCR primer for *Ca SCR1*
ONC206	5'-CAGTCGATGGCAGTTACTTCATG-3'	Fwd RT PCR primer for *FRP1*+1543 to +1565 w.r.t. ATG
ONC207	5'-CACCCTTCTAGCACTTTGACAATG-3'	Rev RT PCR primer for *FRP1* +1623 to +1600 w.r.t. ATG
ONC240	5’-ACCCGAAAGAAAAGTGGTCTTG-3’	Fwd RT ChIP PCR primer at *FRP1* promoter
ONC241	5’-GCATTCAATAAACCTTATTATCATCAGAC-3’	Rev RT ChIP PCR primer at *FRP1* promoter
ONC305	5'-TACCAAGGAACACCAATATCTAGTCACT-3'	Fwd RT ChIP PCR primer at Non-coding seq
ONC306	5'-GATCACTTTTGTCCTTGGCAGTC-3'	Rev RT ChIP PCR primer at Non-coding seq
ONC500	5’-TTTTTTGCTTGCAAAGAGAGAAAA-3’	Fwd RT ChIP PCR primer at *ACO1* -317 to -340 w.r.t. ATG
ONC501	5’-ATCGGATCCATAAAATGGACAAG-3’	Rev RT ChIP PCR primer at *ACO1* -201 to -223 w.r.t. ATG
ONC 911	5'-AATTATTATTCCCACCACACGACAC-3'	Fwd RT ChIP PCR primer for Sef1/CCAAT site at *FTR1*
ONC 912	5'-GATTAGGCCCGATGTACATTGTC-3'	Rev RT ChIP PCR primer for Sef1/CCAAT site at *FTR1*
ONC 921	5'-CGGTGATATCGCTTGGACGTAT-3'	Fwd RT ChIP PCR primer for CCAAT site at *CYC1*
ONC 922	5'-GACGTGACGTGACGAAGAAAGA-3'	Rev RT ChIP PCR primer for CCAAT site at *CYC1*
ONC 927	5'-CCAAGTTCGGAAATGAAACCTAGT-3'	Fwd RT ChIP PCR primer for CCAAT site at *SIT1*
ONC 928	5'-AATTTATTGGCCGCAATCGTA-3'	Rev RT ChIP PCR primer for CCAAT site at *SIT1*
ONC1003	5’-CCCAGCCAATTGTCTTGAAGTT-3’	Forward Primer for PolII/H3-Lys9 Acetylationat *ACO1*-100 to +100wrt ATG
ONC1004	5'-AACCACGAACTGAACGTGGG -3'	Reverse Primer for PolII/H3-Lys9 acetylationat *ACO1*-100 to +100wrt ATG
ONC1006	5'-GGTTTATTGAATGCATGAATGAAAAG-3'	Forward Primer for PolII/H3-Lys9 acetylation at *FRP1*-100 to +100wrt ATG
ONC1007	5'-CCTTTTCCACAAAAAACTGTTGATC-3'	Reverse Primer for PolII/H3-Lys9 acetylationat *FRP1*-100 to +100wrt ATG

Table S4. Key reagents used

Reagent	Cat. No.	Source
Phusion HF DNA polymerase	F-530S	ThermoFisher Scientific
IgG sepharose 6 Fast Flow	17-0969-01	GE Healthcare
Dynabeads Protein G	10004D	ThermoFisher Scientific
Protein G Sepharose 4 Fast Flow	17-0618-01	GE Healthcare
Protran 0.45μM Nitrocellulose Membrane	10600003	GE Healthcare
Power SYBR Green PCR Master Mix	4367659	Applied biosystems
AcTEV protease	12575-015	Invitrogen
MultiScribe Reverse Transcriptase	4319983	Applied biosystems
DNase I	D5307	Sigma
ClonNAT (Nourseothricin)	96736-11-7	Werner Bioagents Gmbh
anti-Hap43 antibody	(in-house)	(1)
anti-TAP antibody	CAB1001	ThermoFisher Scientific
anti-FLAG antibody	F1804	Sigma-Aldrich (Merck)
anti-G6PDH antibody	A9521	Sigma-Aldrich (Merck)
anti-histone H3 Lys9-Ac antibody	07-352	Merck Millipore
anti-histone H3 antibody	Ab1791	Abcam
anti-rabbit HRP conjugate secondary antibody	NA934VS	GE Healthcare
anti-mouse HRP conjugate secondary antibody	NIF825	GE Healthcare
anti-RNA polymerase II antibody	ab5408	Abcam

References

1. Singh RP, Prasad HK, Sinha I, Agarwal N, & Natarajan K (2011) Cap2-HAP Complex Is a Critical Transcriptional Regulator That Has Dual but Contrasting Roles in Regulation of Iron Homeostasis in *Candida albicans*. *J. Biol. Chem.* 286(28):25154-25170.
2. Noble SM & Johnson AD (2005) Strains and strategies for large-scale gene deletion studies of the diploid human fungal pathogen Candida albicans. *Eukaryot. Cell* 4(2):298-309.
3. Sinha I, Kumar S, Poonia P, Sawhney S, & Natarajan K (2017) Functional Specialization of Two Paralogous TAF12 Variants by Their Selective Association with SAGA and TFIID Transcriptional Regulatory Complexes. *J. Biol. Chem.* 292(15):6047–6055.

4. Sharma S, *et al.* (2014) Sphingolipid biosynthetic pathway genes *FEN1* and *SUR4* modulate amphotericin B resistance. *Antimicrob. Agents Chemother.* 58(4):2409-2414.
5. Uhl MA & Johnson AD (2001) Development of Streptococcus thermophilus lacZ as a reporter gene for Candida albicans. *Microbiology* 147(Pt 5):1189-1195.
6. Murad AM, Lee PR, Broadbent ID, Barelle CJ, & Brown AJ (2000) CIp10, an efficient and convenient integrating vector for Candida albicans. *Yeast* 16(4):325-327.
7. Baek YU, Li M, & Davis DA (2008) *Candida albicans* ferric reductases are differentially regulated in response to distinct forms of iron limitation by the Rim101 and CBF transcription factors. *Eukaryot. Cell* 7(7):1168-1179.
8. Chen C, Pande K, French SD, Tuch BB, & Noble SM (2011) An iron homeostasis regulatory circuit with reciprocal roles in *Candida albicans* commensalism and pathogenesis. *Cell Host Microbe* 10(2):118-135.
9. Moehle CM & Hinnebusch AG (1991) Association of RAP1 binding sites with stringent control of ribosomal protein gene transcription in *Saccharomyces cerevisiae*. *Mol. Cell. Biol.* 11:2723-2735.
10. Natarajan K, Jackson BM, Rhee E, & Hinnebusch AG (1998) $yTAF_{II}61$ has a general role in RNA polymerase II transcription and is required by Gcn4p to recruit the SAGA coactivator complex. *Mol. Cell* 2:683-692.
11. Wu PY, Ruhlmann C, Winston F, & Schultz P (2004) Molecular architecture of the S. cerevisiae SAGA complex. *Mol. Cell* 15(2):199-208.
12. Livak KJ & Schmittgen TD (2001) Analysis of relative gene expression data using real-time quantitative PCR and the 2^{-DDCT} method. *Methods* 25(4):402-408.
13. Reuss O, Vik A, Kolter R, & Morschhauser J (2004) The SAT1 flipper, an optimized tool for gene disruption in Candida albicans. *Gene* 341:119-127.
14. Gola S, Martin R, Walther A, Dunkler A, & Wendland J (2003) New modules for PCR-based gene targeting in Candida albicans: rapid and efficient gene targeting using 100 bp of flanking homology region. *Yeast* 20(16):1339-1347.
15. Gillum AM, Tsay EY, & Kirsch DR (1984) Isolation of the Candida albicans gene for orotidine-5'-phosphate decarboxylase by complementation of S. cerevisiae ura3 and E. coli pyrF mutations. *Mol. Gen. Genet.* 198(1):179-182.
16. Homann OR, Dea J, Noble SM, & Johnson AD (2009) A phenotypic profile of the *Candida albicans* regulatory network. *PLoS Genet.* 5(12):e1000783.

Figure Legends

Figure S1. Iron deprivation induced Hap3 and Sef1 protein expression in SC-LIM medium. Protein extracts prepared from TAP-tagged strains RPY453 (Hap5), KNC398(Hap2), KNC410 (Hap3) and KNC379 (Sef1) under iron replete and deplete conditions in SC-LIM media were separated on 10% SDS-PAGE and probed with α-TAP and α-G6PDH as loading control. *A*, Western blot analysis of TAP-tagged proteins in +Fe and -Fe.and the western blot images were quantified, normalized to G6PDH levels and plotted. ***C***. The error bars represent SEM (n=4), and statistical significance was determined by Student's T-test, and P-value ≤0.05 was considered statistically significant and indicated as P≤0.05 (*) and P ≤0.001 (***).

Figure S2. *HAP43* expression is partially dependent on Sef1. *A*, Expression of *HAP43* promoter fused to *lac*Z reporter gene. β-galactosidase activity was assayed in cell extracts from WT (KNC332), *hap2Δ/Δ* (KNC340) and *sef1Δ/Δ* (KNC348) strains. The error bars represent SEM (n=4), and a P-value ≤0.05 was considered statistically significant indicated as p-value ≤0.01 (**), p-value ≤0.001 (***) and p-value ≤0.0001 (****). *B, HAP43* mRNA level is strongly reduced in *sef1*Δ/Δ mutant. *C*, Hap43 protein level is strongly impaired but not lost in the *sef1*Δ/Δ mutant. Protein extracts from WT and *sef1Δ/Δ* strains were grown under -Fe conditions and 150μg, 75μg, 37.5μg and 18.75μg total protein was loaded on 10% SDS-PAGE and transferred to Protran nitrocellulose membrane. Hap43 protein was detected by affinity-purified α-Hap43 antibody, and α-G6PDH antibody was used as loading control. *D*, Hap43 protein levels in wild type and *sef1*Δ/Δ were quantified using ImageJ software and plotted.

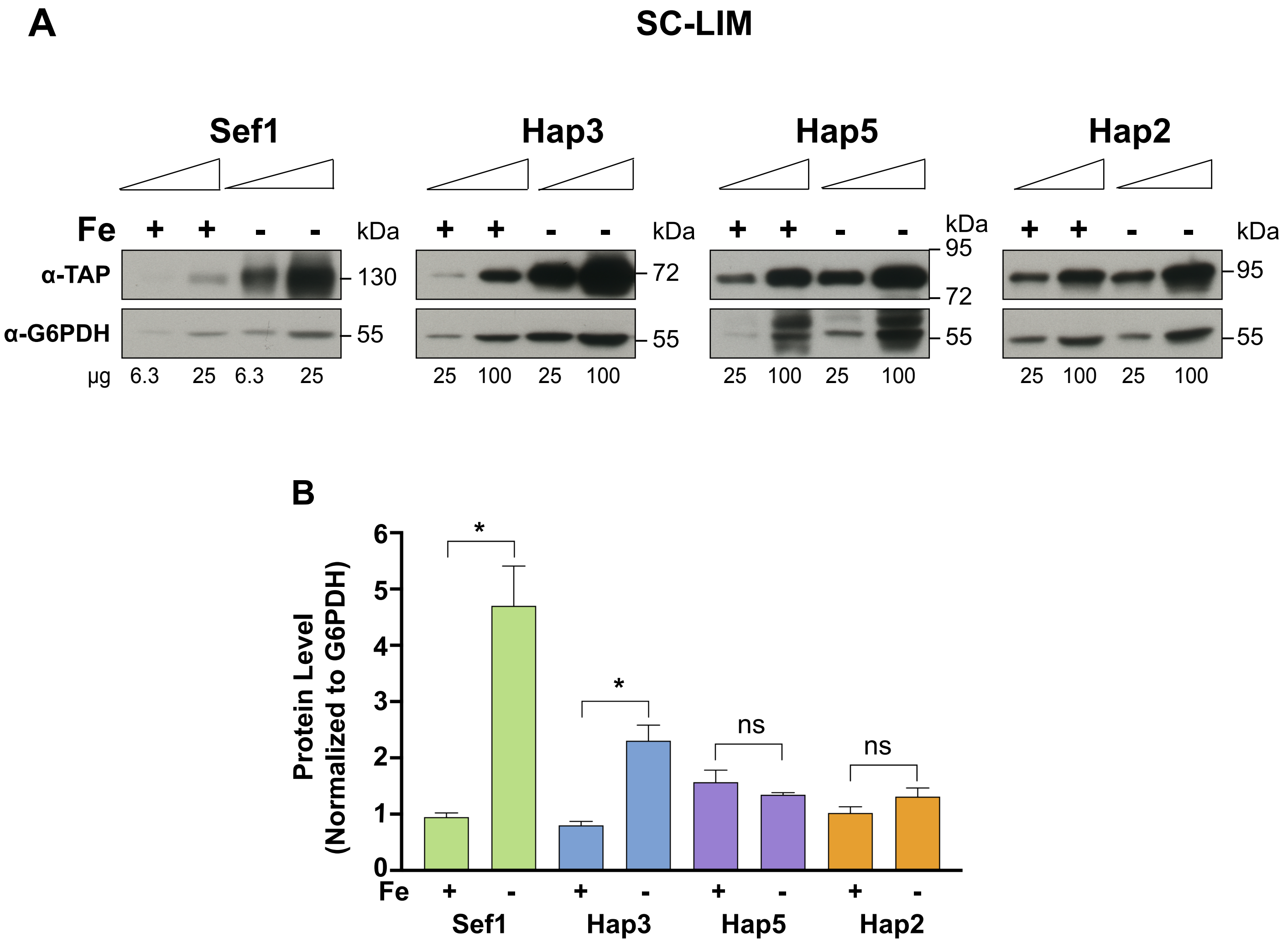
A
SC-LIM
Sef1
Hap3
Hap5
Hap2
Fe + + - -
kDa
α-TAP
α-G6PDH
130
55
72
55
95
72
55
95
55
μg 6.3 25 6.3 25
25 100 25 100
25 100 25 100
25 100 25 100
B
Protein Level
(Normalized to G6PDH)
0
1
2
3
4
5
6
*
*
ns
ns
Fe + - + - + - + -
Sef1
Hap3
Hap5
Hap2

Fig.S1

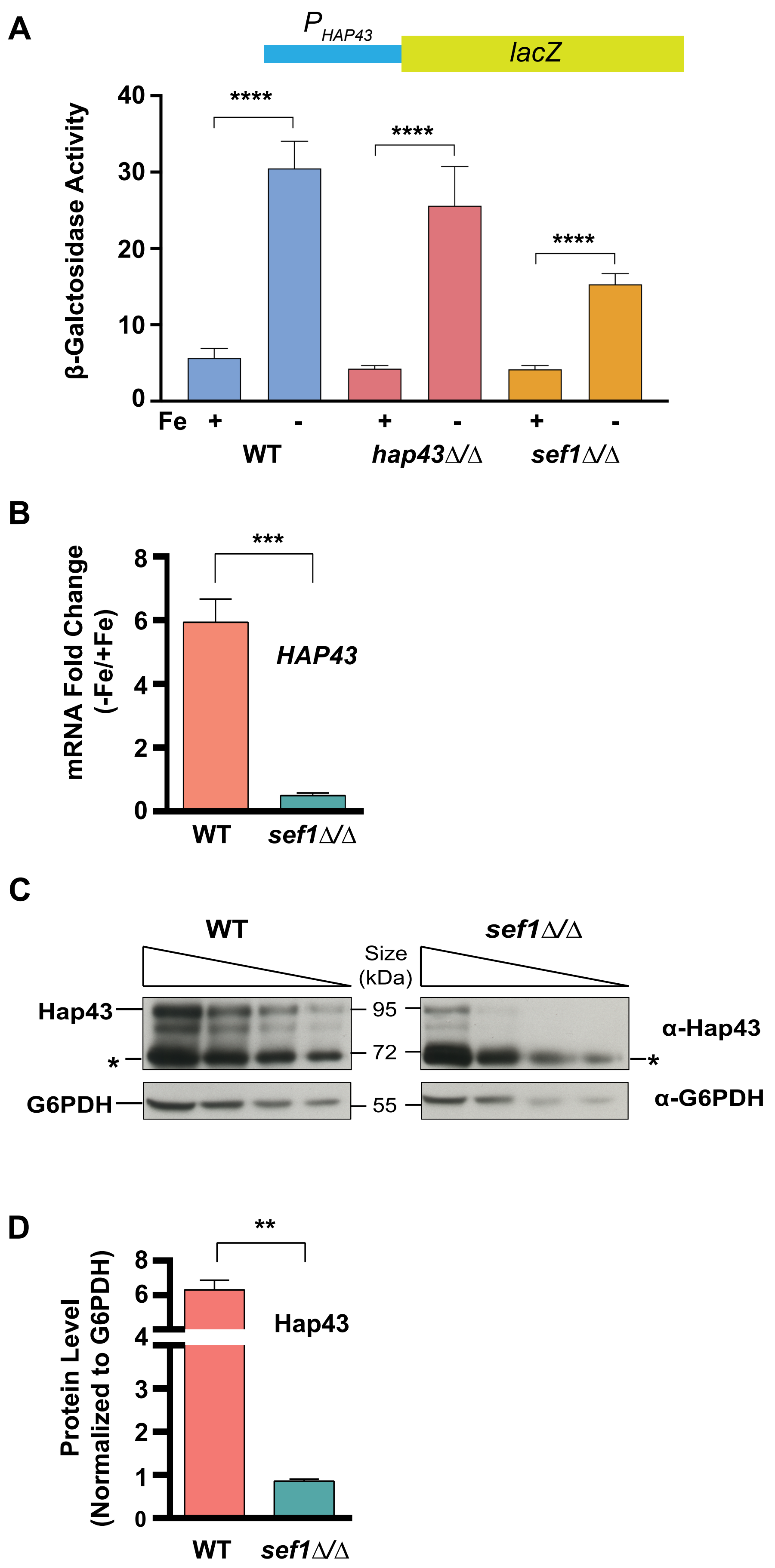
A
P_{HAP43}
lacZ
β-Galctosidase Activity
40
30
20
10
0
****
****
****
Fe + - + - + -
WT
hap43Δ/Δ
sef1Δ/Δ
B
mRNA Fold Change
(-Fe/+Fe)
8
6
4
2
0
***
HAP43
WT
sef1Δ/Δ
C
WT
sef1Δ/Δ
Size
(kDa)
Hap43
*
95
72
55
α-Hap43
*
G6PDH
α-G6PDH
D
Protein Level
(Normalized to G6PDH)
8
6
4
3
2
1
0
**
Hap43
WT
sef1Δ/Δ

Fig. S2
